# Comparing divisome organization between vegetative and sporulating *Bacillus subtilis* at the nanoscale using DNA-PAINT

**DOI:** 10.1126/sciadv.adk5847

**Published:** 2024-01-10

**Authors:** Kimberly Cramer, Susanne C. M. Reinhardt, Alexander Auer, Jae Yen Shin, Ralf Jungmann

**Affiliations:** ^1^ Faculty of Physics and Center for Nanoscience, Ludwig Maximilian University, Munich, Germany.; ^2^ Max Planck Institute of Biochemistry, Martinsried, Germany.

## Abstract

Spore-forming bacteria have two distinct division modes: sporulation and vegetative division. The placement of the foundational division machinery component (Z-ring) within the division plane is contingent on the division mode. However, investigating if and how division is performed differently between sporulating and vegetative cells remains challenging, particularly at the nanoscale. Here, we use DNA-PAINT super-resolution microscopy to compare the 3D assembly and distribution patterns of key division proteins SepF, ZapA, DivIVA, and FtsZ. We determine that ZapA and SepF placement within the division plane mimics that of the Z-ring in vegetative and sporulating cells. We find that DivIVA assemblies differ between vegetative and sporulating cells. Furthermore, we reveal that SepF assembles into ~50-nm arcs independent of division mode. We propose a nanoscale model in which symmetric or asymmetric placement of the Z-ring and early divisome proteins is a defining characteristic of vegetative or sporulating cells, respectively, and regulation of septal thickness differs between division modes.

## Teaser

The division mode of spore-forming bacteria dictates the distinct organization and placement of the cell division machinery.

## INTRODUCTION

**Fig. 1.**
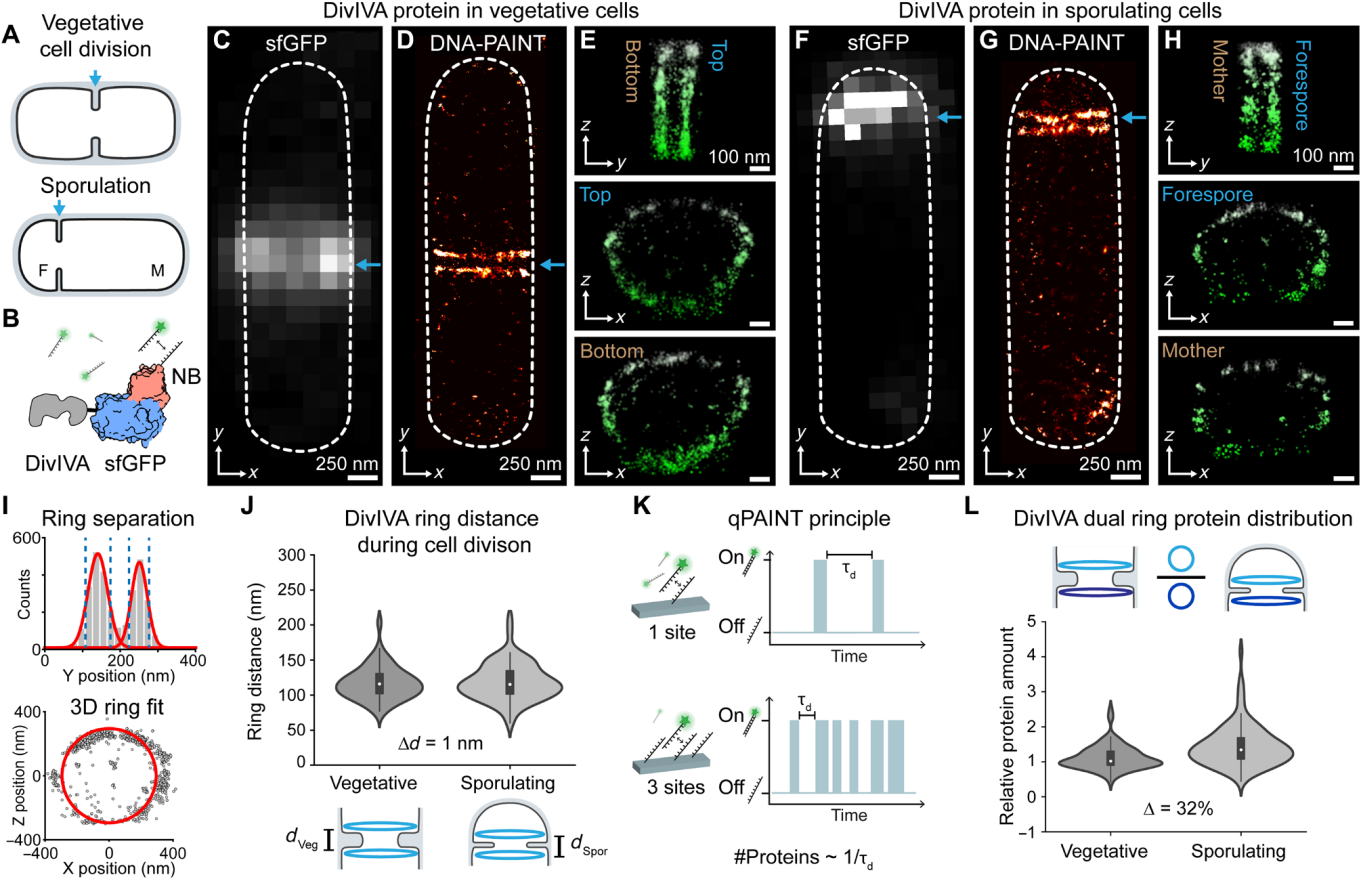
Geometric and stoichiometric analysis of DivIVA at the division plane in vegetative and sporulating cells. (**A**) Cell division, blue arrows, occurs at mid-cell during vegetative division and asymmetrically during sporulation. Sporulating cells contain a smaller forespore (F) and a larger mother cell (M) compartment. (**B**) Labeling schematic of DivIVA-sfGFP with DNA-conjugated nanobody (NB). (**C** to **E**) DivIVA localization in a vegetative cell (arrows). (C) Diffraction-limited super-folder green fluorescent protein (sfGFP) imaging shows one band of DivIVA at mid-cell. (D) DNA-PAINT reveals that DivIVA forms two distinct bands. (E) Top: The *zy* projection of DivIVA reveals distinct bands. Middle and bottom: *zx* views unveil DivIVA as two separate rings. (**F** to **H**) DivIVA localization in a sporulating cell (arrows). (**I**) Analysis framework extracting ring-to-ring distances (top) and ring radii (bottom). (**J**) Distance between DivIVA rings in dividing vegetative (*d*_Veg_) and sporulating (*d*_Spor_) cells. Median values in vegetative and sporulating cells were 116 nm (IQR 103 to 131 nm, *n* = 53) and 115 nm (IQR 101 to 145 nm, *n* = 64), respectively, indicating *d*_Veg_ and *d*_Spor_ are similar. Medians are not significantly different (Mood’s test). (**K**) qPAINT principle stating protein amount correlates with 1/τ_d_. One docking site (on one protein) exhibits a certain time, τ_d_, between binding events while three docking sites (on three proteins) yield three times shorter τ_d_. (**L**) DivIVA dual ring protein distribution comparison between cell types. Median values in vegetative and sporulating cells were 1.02 (IQR 0.89 to 1.29, *n* = 97) and 1.34 (IQR 1.09 to 1.69, *n* = 98), respectively. Medians are significantly different (Mood’s test), *P* = 8.5 × 10^−6^. Thus, the DivIVA protein amount is similar in rings in vegetative cells but in sporulating cells, the DivIVA ring in the forespore contains more protein. Plots (J) and (L): Boxes represent IQR (interquartile range), 25 and 75% data quartiles. Whiskers show 1.5 × IQR. White dots indicate median values.

Bacterial cell division is a fundamental process in which a complex macromolecular protein machine, the divisome, splits the cell in two. *Bacillus subtilis*, the gram-positive model organism, exhibits two distinct modes of cell division: vegetative and sporulation. Vegetative cells divide symmetrically at the mid-cell to produce two daughter cells. During sporulation, the cell divides asymmetrically near a single-cell pole, generating a larger mother cell compartment and a smaller compartment called the forespore (Fig. 1A). In both division modes, the tubulin homolog FtsZ assembles into filaments that create a ring-like structure, the Z-ring, at the future division site (*1*). The Z-ring forms a scaffold upon which tens of other proteins assemble, forming the divisome (*2*).

In *B. subtilis*, divisome assembly occurs in two steps (*3*). The first step is an assembly of so-called “early proteins” that bind and support Z-ring formation at the division site (*4*–*6*). The early divisome protein ZapA stabilizes the Z-ring by acting as a protein cross-linker between FtsZ filaments (*7*). Early proteins FtsA and SepF promote Z-ring formation by anchoring FtsZ filaments to the membrane using an amphipathic alpha helix (*5*, *8*, *9*). SepF is widely conserved across not only gram-positive bacteria but also archaea and cyanobacteria (*10*). In vitro, SepF polymers form regular rings with 50-nm diameters that can bundle FtsZ filaments. SepF ring diameter both correlates with and controls septal thickness in vegetatively dividing bacteria (*11*). However, it is unlikely that SepF forms 50-nm rings in cells since the N-terminal membrane-binding domain is located inside the ring (*5*). Therefore, SepF was hypothesized to form arcs encircling the invaginating membrane (*5*, *11*). In the second divisome assembly step, “late proteins” arrive after Z-ring assembly and are involved in cell wall synthesis or remodeling and divisome regulation (*3*).

A major difference in divisome organization between vegetative and sporulating cells is the localization of the Z-ring and FtsA within the division plane. FtsA and FtsZ were found to be symmetrically placed at constricting septa in vegetative cells and asymmetrically placed in the mother cell compartment in sporulating cells (*12*). Whether other early divisome proteins also localize asymmetrically with the Z-ring is not yet determined. The protein DivIVA distributes differently across the division plane dependent on division mode (*13*). DivIVA is the localizer of *B. subtilis* Min system proteins, which prevent aberrant Z-ring assembly and help determine the division site (*14*–*16*). Thus, it is likely that regulatory proteins also assemble differently between division modes. Further knowledge about cell type–dependent differences in the three-dimensional (3D) nanoscale assemblies and localizations of division proteins and their regulators remain sparse. Super-resolution microscopy studies visualizing divisome proteins in sporulating cells remain relatively unexplored (*13*, *17*). However, super-resolution approaches could help understand divisome organization by providing insights into the underlying mechanisms governing division during vegetative growth and sporulation and help reveal if mechanisms differ between division modes.

Here, we use DNA-PAINT super-resolution microscopy (*18*) in combination with quantitative analyses to map the nanoscale cellular localization and spatial arrangement of several cell division proteins and determine potential differences in cell division machinery between vegetative and sporulating *B. subtilis*. We reveal that the Z-ring anchoring protein SepF forms arcs and measure similar SepF arc diameters in both cell types, indicating that SepF arcs do not regulate septal thickness in sporulating cells. We determine DivIVA ring assemblies differ between cell types. In addition, we map the positioning of the Z-ring, SepF, ZapA, and DivIVA rings at division septa and compare ring protein amounts between cell types. Together, our results show the asymmetric placement of divisome proteins toward the mother cell compartment as a key characteristic of sporulating cells and strongly suggest septal thickness regulation differs between division modes.

## RESULTS

### Geometric and stoichiometric analysis of DivIVA at the division plane in vegetative and sporulating cells

First, to investigate regulation of Z-ring placement, we determined the nanoscale arrangement of DivIVA in sporulating and vegetative cells, which might suggest different localization of the Min system. To this end, we set out to characterize the 3D assembly of DivIVA, which serves as a localizer for Min system proteins to the division site (*14*, *15*). To visualize DivIVA in individual cells, we developed a protocol to image bacterial proteins with DNA-PAINT super-resolution microscopy (see Materials and Methods). Briefly, for DNA-PAINT, an oligonucleotide (docking strand) is conjugated to a target binder (e.g., antibody) and its complementary dye-labeled oligonucleotide (imager strand) diffuses through the solution. When these transiently bind, a blinking signal is created, captured by a microscope, and rendered as a super-resolution image (*18*). To implement DNA-PAINT, we first constructed a *B. subtilis* strain (KCB300) that expresses DivIVA protein fused to the super-folder green fluorescent protein (sfGFP) from the DivIVA native locus (fig. S1 and table S1). DivIVA-sfGFP localized at the division septa in vegetative and sporulating cells (Fig. 1, C and F), indicating that sfGFP did not affect the location of DivIVA. Next, we used DNA-conjugated GFP nanobodies for DNA-PAINT imaging (Fig. 1B). DNA-PAINT data revealed that DivIVA localized as two distinct bands at the division plane of vegetative cells (Fig. 1D) and organized as dual rings when visualized in 3D (Fig. 1E), agreeing with previous results (*13*). Notably, we report that the 3D dual ring assembly of DivIVA is retained at the polar division site in sporulating cells (Fig. 1, G and H). In both cell types, DivIVA rings localize on opposite sides of the septum (fig. S2). Thus, dual DivIVA rings flank the division septum in vegetative and sporulating cells.

To further investigate DivIVA dual rings in individual cells, we developed an image analysis framework (see Materials and Methods). Briefly, after interactive selection in the Picasso software (*18*), an automated fitting procedure first separates the DivIVA rings in an *xy* projection using two-component Gaussian fits with appropriate thresholds (see Materials and Methods and Fig. 1I). Next, a 3D ring fit extracts ring properties for analysis. Using this analysis framework, parameters such as the distance between the dual rings, their radii, and further quantitative DNA-PAINT characteristics can be extracted. We first determined the distance between DivIVA dual rings in individual vegetative and sporulating *B. subtilis* undergoing division (i.e., the Z-ring was also present) and calculated median values of 116 nm [interquartile range (IQR) 103 to 131 nm] and 115 nm (IQR 101 to 145 nm), respectively (Fig. 1J). Thus, the distance between DivIVA dual rings is similar between dividing vegetative and sporulating cells.

Previous work showed DivIVA localized in a biased manner across the division plane in sporulating cells, with more DivIVA present in the forespore compartment (*13*). Considering our newfound assembly of DivIVA into dual rings (Fig. 1H), we wanted to quantify if more DivIVA protein is present in the DivIVA ring assembling within the forespore. We used qPAINT (*19*), which is a method to assess protein amounts via DNA-PAINT. In qPAINT, the time between two imager binding events, called dark time τ_d_ (Fig. 1K), inversely scales with the amount of protein present in rings (#proteins ~1/τ_d_). Using this relation, we compared the relative protein amounts between both rings for vegetative and sporulating cells (Fig. 1L). We found a median ratio of 1.02 (IQR 0.89 to 1.29) for vegetative cells and 1.34 (IQR 1.09 to 1.69) for sporulating cells, respectively, indicating a higher protein amount in the forespore DivIVA ring in sporulating cells (Fig. 1L). This suggests reduced localization of the Min system in the mother cell compartment where the asymmetric Z-ring is placed.

### Z-ring placement between dual DivIVA differs between cell types

**Fig. 2.**
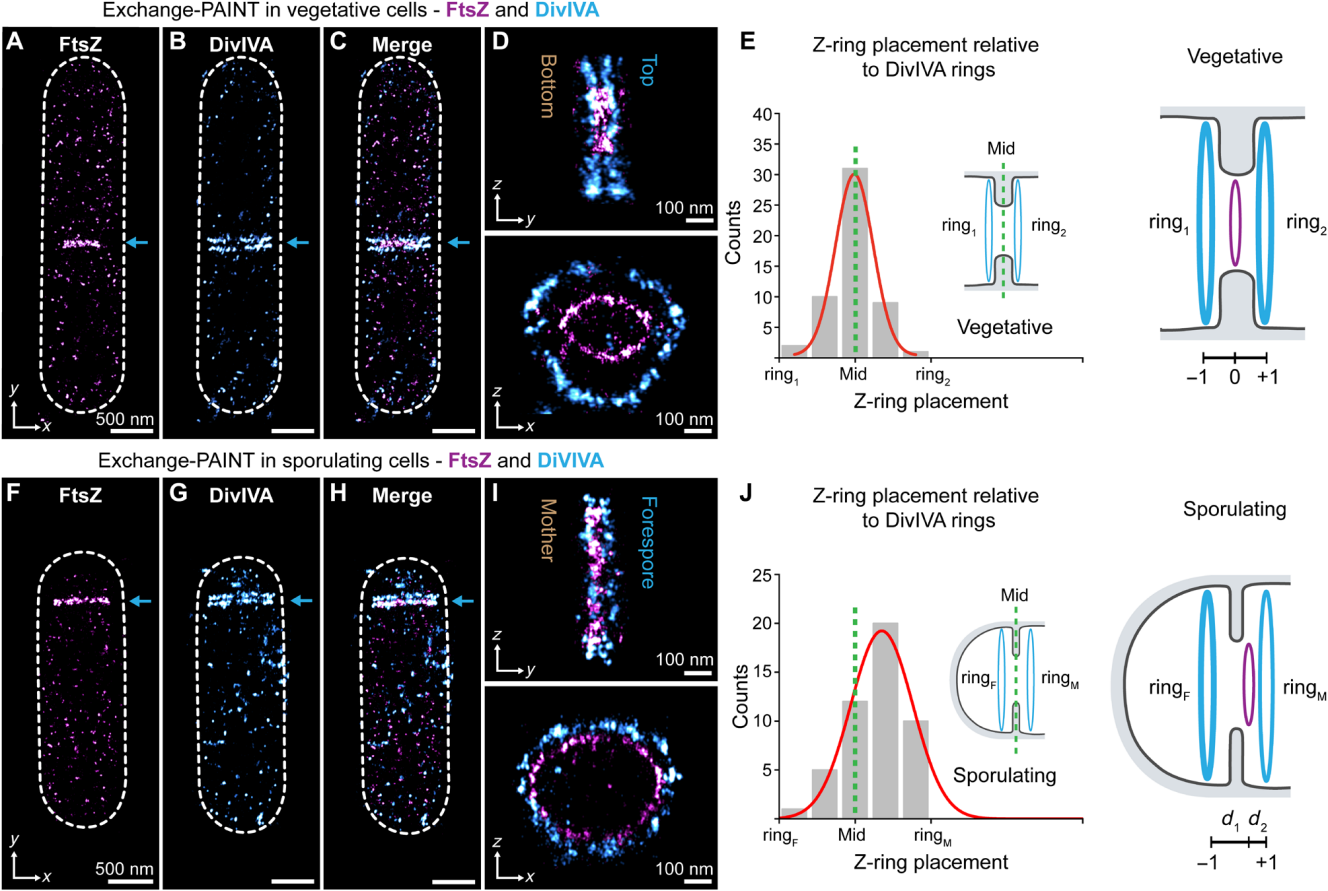
Z-ring placement relative to DivIVA dual rings differs between cell types. Two-target Exchange-PAINT imaging of FtsZ (magenta) and DivIVA (cyan) in dividing vegetative (**A** to **D**) and sporulating (**F** to **I**) *B. subtilis.* [(A) and (F)] FtsZ localization. [(B) and (G)] DivIVA localization. [(C) and (H)] Merged Exchange-PAINT image of an entire cell. Arrows point to protein localization at division sites. [(D) and (I)] Top: The *zy* view of DivIVA and FtsZ. Bottom: The *zx* view of DivIVA and Z-ring “sandwich” arrangement showing the slightly smaller diameter of the (constricting) Z-ring compared to DivIVA. (**E**) Distribution of Z-ring position relative to dual DivIVA rings in vegetative cells. ring_1_ and ring_2_ are DivIVA rings in separate daughter cells. A green dotted line marks the center point between DivIVA rings. Corresponding Gaussian fit yields 0.0 ± 0.2 nm (means ± SD, *n* = 53, 0 marks a centered Z-ring position with ±1 corresponding to the DivIVA ring positions) revealing that the Z-ring is positioned at the center point between DivIVA rings. (**J**) Distribution of Z-ring position relative to dual DivIVA rings in sporulating cells. A green dotted line marks the center point between DivIVA rings. ring_F_ and ring_M_ are DivIVA rings in the forespore or mother cell compartment, respectively. Gaussian fit yields 0.3 ± 0.3 (means ± SD, *n* = 64, again 0 marks a centered Z-ring position with ±1 corresponding to the DivIVA ring positions), revealing that the Z-ring is positioned toward the DivIVA ring in the mother cell compartment. *d*_1_ (71 ± 38 nm, means ± SD, *n* = 64) and *d*_2_ (48 ± 33 nm, means ± SD, *n* = 64) correspond to different distances between a DivIVA ring and the Z-ring. Scale bars, 500 nm [(B), (C), (G), and (H)].

To test this hypothesis, we then wanted to determine the proximity of the Z-ring to the DivIVA dual rings and, by extension, the Min system. Close proximities would suggest increased probabilities of interaction between the Min system and Z-ring. We were especially curious about sporulating cells, in which the Z-ring is placed toward the mother cell compartment (*12*). To visualize the Z-ring together with DivIVA in individual cells, we implemented two-target Exchange-PAINT (*20*). A combination of a primary antibody anti-FtsZ and DNA-conjugated secondary nanobody allowed us to visualize the Z-ring (fig. S3). We visualized DivIVA as before (Fig. 1). DNA-PAINT revealed two bands of DivIVA sandwiching one FtsZ band at the division plane in both vegetative and sporulating cells (Fig. 2, A to C and F to H). The *zy* and *zx* views clearly show that FtsZ localizes as a ring (Z-ring) flanked by a DivIVA ring on each side in vegetative (Fig. 2D) and sporulating cells (Fig. 2I).

To determine where DivIVA dual rings localize relative to the Z-ring, we quantified the location of DivIVA rings and the FtsZ ring in the *xy* plane using two- and one-component Gaussian fits, respectively (Fig. 2, E and J). In vegetatively dividing cells, we defined −1, 0, and +1 as the position of the first DivIVA ring (ring_1_), the mid-septum, and the second DivIVA ring (ring_2_), respectively. Notably, in vegetative cells, we found the Z-ring is placed at the midpoint between the dual DivIVA rings, or equidistant from each ring (mean position 0.0 ± 0.2) (Fig. 2E). In sporulating cells, we defined −1, 0, and +1 as the position of the DivIVA ring in the forespore compartment (ring_F_), the mid-septum, and the DivIVA ring in the mother cell compartment (ring_M_), respectively. We determined that the Z-ring is placed closer to the DivIVA ring in the mother cell compartment (mean position: 0.3 ± 0.3) (Fig. 2J). In sporulating cells, the Z-ring is on average 48 ± 33 nm (*d*_2_) away from ring_M_ and 71 ± 38 nm (*d*_1_) away from ring_F_. Therefore, the Z-ring localizes approximately 25 nm closer to the DivIVA ring in the mother cell.

### Nanoscale positioning of SepF and ZapA rings relative to the Z-ring does not change between cell types

We then turned our attention to the relative placement of ZapA and SepF to the Z-ring, as both proteins are early divisome components involved in Z-ring stabilization. We were especially curious about the placement of SepF, as current models predict localization on the invaginating septum. However, it stabilizes the Z-ring, which also suggests an asymmetric placement in sporulating cells.

**Fig. 3.**
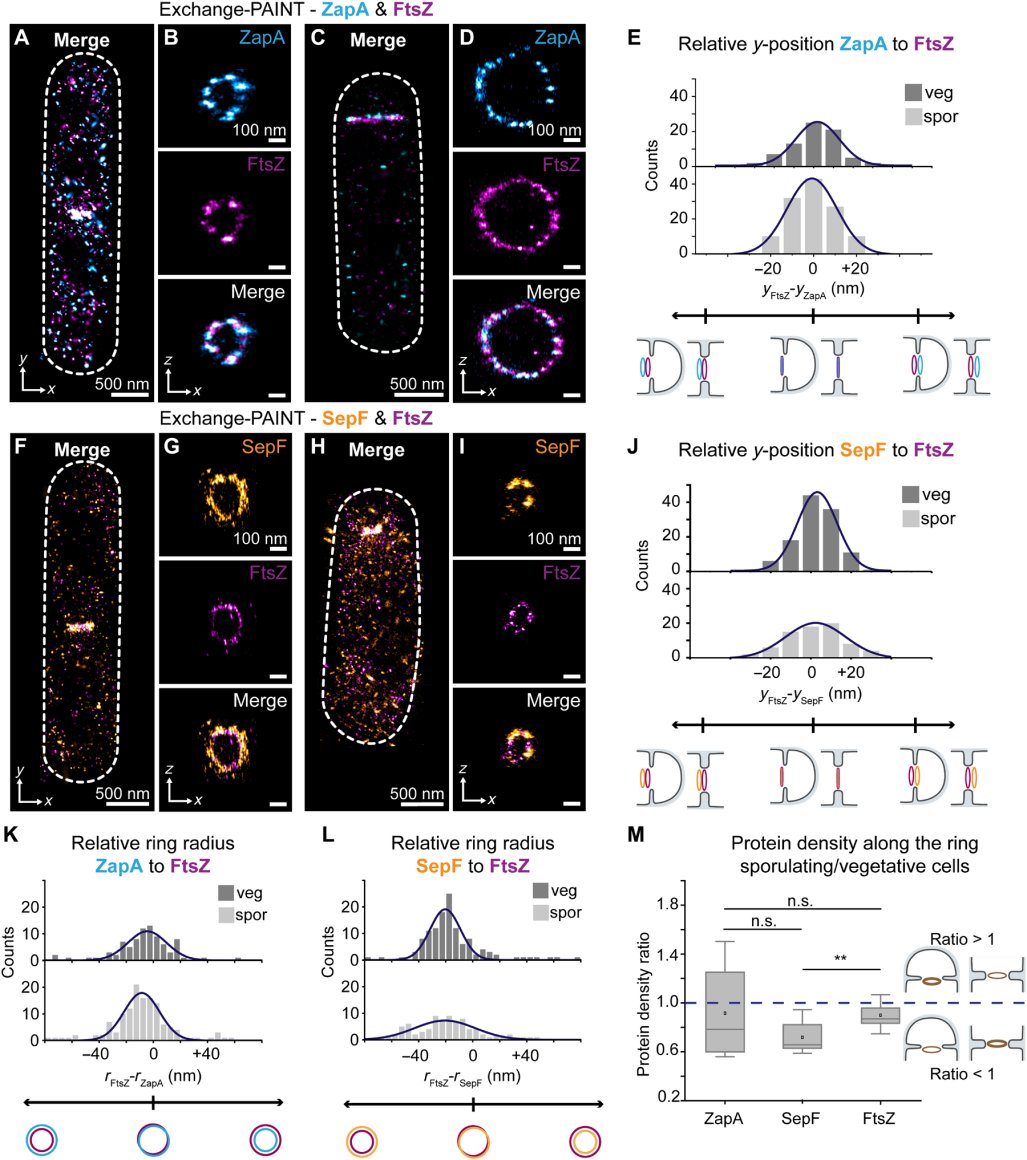
Nanopositioning of SepF and ZapA rings relative to FtsZ. (**A**) Vegetative cells displaying ZapA and FtsZ localization as a band at the mid-cell. (**B**) The *zx* view of ZapA and FtsZ, showing both form rings. (**C** and **D**) Results for a sporulating cell. (**E**) Relative *y*-position distribution of the ZapA ring to the Z-ring in cells shows mean values of 2 ± 11 nm in vegetative and 1 ± 12 nm in sporulating cells, indicating close positioning of the Z-ring and ZapA ring in both division modes. (**F**) Vegetative cells displaying SepF and FtsZ localization as a band at the mid-cell. (**G**) The *zx* view of SepF and FtsZ, showing both form rings. (**H** and **I**) Respective results in a sporulating cell. (**J**) Relative *y*-position distribution of SepF to the Z-ring shows mean values of 3 ± 10 nm in vegetative and 2 ± 15 nm in sporulating cells, indicating close positioning in both division modes. (**K**) Relative ring radius of ZapA to FtsZ (*r*_FtsZ_-*r*_ZapA_): −5 ± 14 nm in vegetative, −9 ± 13 nm in sporulating. (**L**) SepF to FtsZ (*r*_FtsZ_-*r*_SepF_): −20 ± 11 nm vegetative, −21 ± 22 nm sporulating, showing a larger SepF ring radius than Z-ring. (**M**) Ring density ratios of ZapA, SepF, and FtsZ in vegetative and sporulating cells were compared and visualized as box plots. Mean ratios are 0.9 ± 0.4 for ZapA, 0.7 ± 0.1 for SepF, and 0.9 ± 0.1 for Z-rings. Ratios above 1 indicate higher densities in sporulating cells, while below 1 indicates higher densities in vegetative cells. Statistical tests reveal significant differences for SepF and FtsZ (but not ZapA) from a ratio of 1, and significant differences between Z-ring and SepF, but not between ZapA and SepF or FtsZ. Data from 16 (ZapA and SepF) and 56 (FtsZ) fields of view.

We constructed a *B. subtilis* strain expressing ZapA protein fused to ALFA-tag (*21*) from the ZapA native locus (KCB328; fig. S1 and table S1). DNA-conjugated anti–ALFA-tag nanobodies (NB-ALFA) were used for ZapA visualization. Two-target Exchange-PAINT on the Z-ring and ZapA (Fig. 3, A to D) revealed ZapA assembles in 2D as a band (Fig. 3, A and C) and in 3D as a ring structure (Fig. 3, B and D) that appears to localize with the Z-ring in both vegetative and sporulating cells. We then measured the relative distances between FtsZ and ZapA rings along the *y* axis (see Materials and Methods and Fig. 3E and fig. S4). We determined the ZapA ring typically localized within 2 ± 11 nm and 1 ± 12 nm of the Z-ring at vegetative and sporulating division septa, respectively (Fig. 3E). Thus, ZapA rings are positioned together with the Z-ring in both division modes.

We visualized SepF in vegetative and sporulating cells using a bacterial strain expressing the fusion protein SepF-sfGFP from the endogenous loci (strain KCB1113; fig. S1 and table S1) and DNA-conjugated anti-GFP nanobodies to target SepF-sfGFP. SepF was resolved as a band (Fig. 3, F and H) in 2D and as a ring in 3D (Fig. 3, G and I) at division sites in both vegetative and sporulating cells and appeared to localize with the Z-ring. In the same manner, as described above (Fig. 3E), we analyzed the positioning of SepF rings to the Z-ring. SepF was typically localized within 3 ± 10 nm and 2 ± 15 nm of the Z-ring at vegetative and sporulating division septa, respectively (Fig. 3J). Thus, our data show that the SepF ring localizes with the Z-ring at division septa. To further verify SepF and Z-ring colocalization, we sliced our multiplexed data into ~40-nm sections in the *z* direction. At the cellular cross section of dividing vegetative and sporulating cells, FtsZ was bordered by or localized in close proximity to SepF (fig. S5). We conclude the nanoscale placement of SepF and ZapA relative to the Z-ring remains the same at vegetative and sporulating division septa despite the asymmetric placement of the Z-ring. Thus, SepF and ZapA are asymmetrically placed at division septa in sporulating cells.

Next, we compared the radii of the Z-ring, ZapA, and SepF rings. Larger or smaller ring radii would suggest rings assemble closer or further from the cell membrane than others, respectively. The radius of the SepF or ZapA rings was subtracted from the Z-ring radius in individual vegetative or sporulating cells. Positive values indicate that ZapA or SepF rings had smaller radii than the Z-ring and negative values indicate that the ZapA or SepF rings had larger radii. We determined that the relative radii of ZapA rings were −5 ± 14 nm and −9 ± 13 nm (means ± SD) in vegetative and sporulating cells, respectively (Fig. 3K). We determined that the difference between the Z-ring and SepF radii was −20 ± 11 nm and −21 ± 22 nm (means ± SD) in vegetative and sporulating cells, respectively (Fig. 3L), indicating that SepF rings have ~20-nm-larger radii than the Z-ring. We suspect the displacement of ZapA relative to the Z-ring to be caused by an offset due to the nanobody and antibody labeling approach. However, the larger displacement of SepF relative to the Z-ring indicates a true radius difference. Together, data determined that the relative radii of the protein rings are similar in both sporulating and vegetative cells, and data strongly suggest that the SepF ring assembles closest to the cell membrane, followed by the ZapA and FtsZ rings.

Next, we determined if the protein density within the Z-ring, SepF, and ZapA rings differed between vegetative and sporulating cells. To this end, as described above (Fig. 1), we used 1/τ_d_ as a proxy for protein amounts. We compared protein amounts in the rings of each cell type within the same field of view (FOV) (see Materials and Methods and fig. S6). A ring density ratio was created to compare protein densities of sporulating cells to vegetative cells. Ring density refers to the protein amount normalized by the ring circumference. When the ring density ratio is smaller than 1, protein density is larger in vegetative cell rings. When the ratio is >1, it is larger in sporulating cells. While the mean ratio of ZapA rings was 0.9 ± 0.4, the data range and spread indicate no clear trend. Contrarily, the Z-ring and SepF rings presented mean values of 0.9 ± 0.1 and 0.7 ± 0.1, respectively. Thus, indicating SepF rings contain fewer proteins (~30%) in sporulating cells than in vegetative cells and the Z-ring contains slightly fewer proteins (~10%) in sporulating cells than in vegetative cells. Hence, the ring protein density of the Z-ring and SepF ring typically differs between vegetative and sporulating cells.

### SepF assembles as arcs at vegetative and sporulating division septa

**Fig. 4.**
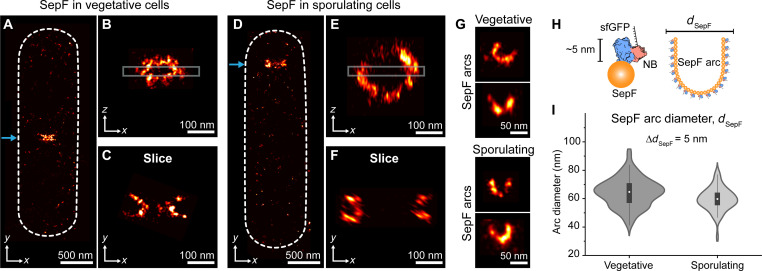
SepF assembles as arcs at vegetative and sporulating division septa. (**A**) DNA-PAINT imaging reveals SepF localizes as a band with “protruding edges” (similar to a half torus) in vegetative cells. The blue arrow points to SepF localization at mid-cell. (**B**) Projection in *zx* of the SepF band in (A) shows that SepF assembles as a ring-like structure. (**C**) Fifty-nanometer slice [as indicated in (B)] shows that SepF forms arcs. (**D** to **F**) Respective results in a sporulating cell. SepF assembles at the asymmetric division site in sporulating cells (blue arrow). (**G**) Further examples of SepF arcs (50-nm slices) in vegetative and sporulating cells. (**H**) Schematic of SepF-sfGFP labeling strategy via DNA-conjugated anti-GFP (sfGFP) NBs. *d*_SepF_, or SepF arc diameter, is defined as the distance between endpoints of SepF arcs. NBs face outward when bound to SepF arcs. (**I**) The distribution of SepF arc diameters, *d*_SepF_, in each division mode is shown as violin plots. *d*_SepF_ is 64.7 nm (median, IQR 57.1 to 70.8, *n* = 96) and 59.6 nm (median, IQR 55.5 to 64.1, *n* = 55) for vegetative and sporulating cells, respectively. Boxes represent IQR (interquartile range), 25% and 75% quartile of data. Whiskers show 1.5 × IQR. White dots indicate median values.

We noticed SepF bands exhibiting “protruding edges” in our images. Thus, we next focused our attention on testing the hypothesis that SepF proteins form arcs with diameters correlating with and influencing the division septum thickness in vegetative cells (*11*). SepF-sfGFP and the Z-ring were labeled as previously described (Fig. 3 and fig. S3). Similar to the organization observed in Fig. 3, DNA-PAINT imaging revealed that SepF assembled as a band at the division plane in sporulating and vegetative cells (Fig. 4, A and D) and forms a ring structure in 3D (Fig. 4, B and E). Notably, we noticed the edges of SepF bands consistently protruded into an arc shape. Therefore, we sliced SepF rings into 35- to 55-nm sections in the *z* plane. We then looked at the SepF assembly at the midpoint, the center of the cell in the *z* direction, and found that SepF forms clear arc structures at this cellular cross section (Fig. 4, C and F). We found arcs consistently at the cross section of multiple dividing *B. subtilis* (Fig. 4G), further indicating that SepF rings at division septa are composed of arc-shaped protein assemblies. Since the diameter of SepF protein rings in vitro correlates with division septum thickness, we set out to determine whether the SepF arc diameter corresponded with the reported thickness of the dividing septa in sporulating and vegetative cells. We defined the distance between the two endpoints on SepF arcs as arc diameter, which was measured by determining the distance between the two arc endpoints (see Materials and Methods). The 3D arrangement of SepF-sfGFP in combination with its labeling probe, NB-GFP, at the septum, should be considered for the true SepF arc diameter (Fig. 4H). NB-GFP is 4 to 5 nm long and GFP is fused to the C terminus of SepF. Thus, anti-GFP nanobodies would create a displacement of approximately 5 nm from their binding site to the true SepF position, adding up to 10 nm to the total arc diameter. Distribution centers indicate that typical SepF arc diameter is 64.7 nm (median, IQR 57.1 to 70.8) and 59.6 nm (median, IQR 55.5 to 64.1) for vegetative and sporulating cells, respectively (Fig. 4I). Thus, true SepF arc diameter is likely ~50 nm in both cell types (Fig. 4I), in good agreement with reported septal thickness in vegetative (50 nm) but not sporulating (25 nm) cells (*12*).

## DISCUSSION

**Fig. 5.**
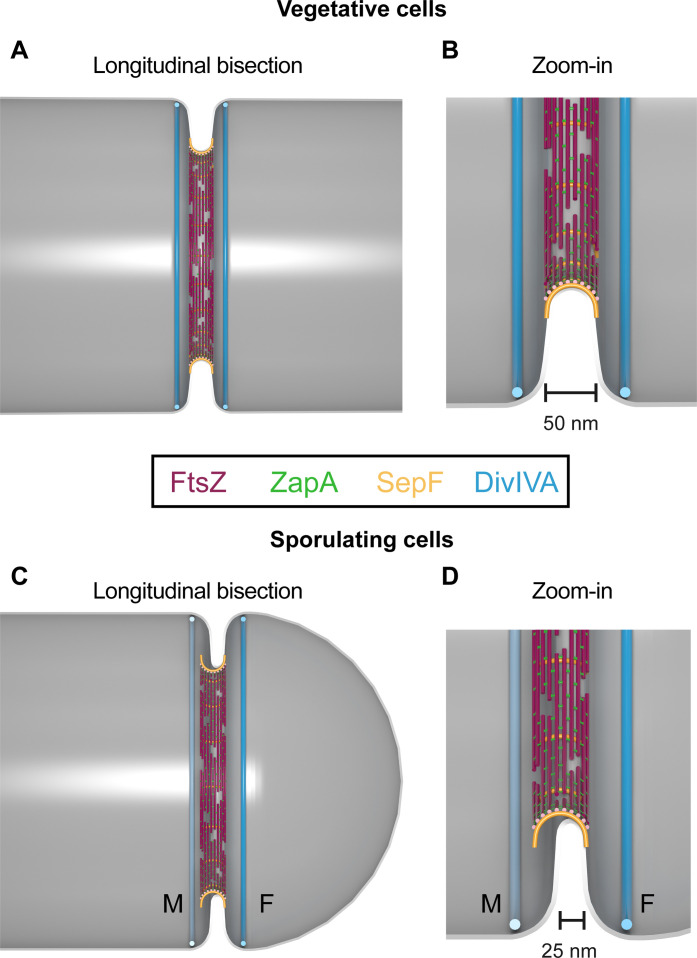
Nanoscale model of SepF, FtsZ (Z-ring), ZapA, and DivIVA 3D assembly and placement within vegetative and sporulating septa. (**A**) Model of divisome protein assembly and placement within a longitudinal bisection of a vegetatively dividing cell. The invaginating septum is approximately 50 nm thick. FtsZ filaments, which form the Z-ring, localize at the septum. SepF assembles as arcs with ~50-nm diameters that encircle the invaginating septum. SepF arcs stabilize and align the FtsZ filaments that lie perpendicularly across them. “Dog bone–shaped” ZapA tetramers stabilize the Z-ring by acting as a protein cross-linker between FtsZ filaments. DivIVA rings sandwich the active divisome, placed equidistant from the Z-ring, preventing aberrant Z-ring formation. DivIVA protein distributes evenly between its dual rings. (**B**) Zoom-in of the bottom portion of the division plane in (A). (**C**) Model of divisome protein assembly and placement within a longitudinal bisection of a sporulating cell. The invaginating septum is approximately 25 nm thick. M is the mother cell compartment. F is forespore. The Z-ring, composed of FtsZ filaments, is placed asymmetrically on the septum toward M and contains 10% less protein than in vegetative cells. SepF and ZapA are asymmetrically placed with the Z-ring. SepF forms 50-nm arcs which cannot perfectly encircle the thinner (25 nm) invaginating septum in sporulating cells. This enables asymmetric localization of SepF arcs toward the M. ZapA cross-links FtsZ filaments. DivIVA rings flank the active divisome. The Z-ring localizes ~25 nm closer to the DivIVA ring in M, enabling quick divisome disassembly for relocation of FtsZ to the division site at the opposite cell pole. DivIVA ring in M has ~30% less protein than its counterpart. (**D**) Zoom-in of the bottom portion of the division plane in (C). FtsZ, magenta; SepF, orange; DivIVA, cyan; ZapA, green. The cell membrane is gray.

On the basis of our findings, we are now able to present a nanoscale model (Fig. 5) for the localization of FtsZ, early divisome proteins, and DivIVA in vegetative and sporulating cells.

In vegetative cells, the Z-ring is placed at the mid-septa. SepF assembles as arcs with ~50-nm diameters (Fig. 4) that encircle and line the invaginating septum (Fig. 3), creating a ring-like structure (Figs. 3 and 4) (*5*, *22*). SepF stabilizes and aligns FtsZ protofilaments that treadmill with associated peptidoglycan synthesis machinery (*23*, *24*), ultimately affecting septal thickness. ZapA tetramers laterally cross-link FtsZ filaments (*4*, *25*) to stabilize the Z-ring at the division site (Fig. 3). After membrane invagination begins, DivIVA rings flank the septum equidistant from the Z-ring (Fig. 2). DivIVA localizes the Min system that functions to inhibit aberrant Z-ring assembly near the division site (*26*).

In sporulating cells, dual DivIVA rings also flank the division site (Fig. 2). The Z-ring is asymmetrically placed ~25 nm closer to the DivIVA ring in mother cell compartment (Fig. 2). Similar to vegetative cells, SepF colocalizes with the Z-ring and assembles as 50-nm arcs (Fig. 3). However, SepF arcs are asymmetrically placed across the thinner (~25 nm) invaginating membrane toward the mother cell compartment (Figs. 3 and 4). Here, SepF arcs stabilize FtsZ protofilaments (*5*, *22*) but do not regulate septal thickness. Again, ZapA tetramers cross-link FtsZ filaments (Fig. 4) to promote its assembly (*7*). In addition, we calculated the longitudinal positioning of the Z-ring, SepF, ZapA, and DivIVA rings relative to cell length in sporulating cells (fig. S7) based on findings in Figs. 2 and 3.

Our results strongly suggest that SepF arcs do not function to regulate septum thickness in sporulating cells. Previous “clamp models” postulated SepF forms arcs that wrap around the leading edge of the constricting septa in vegetative cells (*11*, *22*). These models were derived from experiments involving purified SepF and FtsZ polymers and cryo–electron microscopy of the cell membrane, during which ring-shaped polymers of SepF were observed. We determined that SepF forms arcs in situ (Fig. 4), and the SepF ring localizes closer to the cell membrane than the Z-ring (Fig. 3) and is placed at the same position as the Z-ring within the dividing septum (Fig. 3). Thus, the clamp model of SepF assembly seems extremely likely. We propose that the thickness of the septa is controlled by SepF arc diameter (*11*) in vegetative cells, in which both dividing septum thickness and SepF arc diameter of ~50 nm correlate. We show that arc diameters of ~50 nm (Fig. 4) are present in sporulating cells, which contain a 25-nm-thick septum (*12*). Thus, SepF arc diameter most likely does not regulate septum thickness in sporulating cells. The protein SpoIIE, which is only expressed in sporulating cells and shown to modulate septal thickness (*12*, *27*, *28*), could offer a potential explanation.

Our results indicate that the asymmetric placement of Z-ring stabilizing proteins toward the mother cell compartment is characteristic of sporulating *B. subtilis* (Fig. 3). This was especially interesting because the cell places the Z-ring asymmetrically during sporulation (*12*). Our results are expected for ZapA, which functions as a protein cross-linker between FtsZ filaments (*4*, *29*). However, it was unclear if SepF would colocalize with the Z-ring toward the mother cell compartment (*12*) or at the mid-septum in sporulating cells. While SepF recruitment to the division site depends on FtsZ (*30*), its N terminus binds cellular membranes (*5*) and SepF arcs in vegetative cells localized at the mid-septa (Fig. 3). We show that the SepF ring localizes asymmetrically in sporulating cells. It is possible that SepF interaction with the Z-ring promotes the asymmetric positioning of SepF at the division site. FtsA was also shown to localize with the Z-ring in sporulating cells (*12*). Thus, all known divisome proteins involved in promoting functional Z-ring assembly early in division (SepF, ZapA, and FtsA) assemble asymmetrically with the Z-ring in sporulating cells.

DNA-PAINT imaging revealed both differences and similarities between DivIVA dual rings in division modes (Figs. 1 and 2). We calculated that the DivIVA ring in the forespore (ring_F_) contained ~30% more DivIVA than its counterpart in the mother cell compartment (ring_M_) in dividing cells. A previous study (*13*) reported that DivIVA was present only in the forespore compartment after division. A geometric factor that might promote DivIVA protein localization in ring_F_ is the geometry of the membrane in the forespore compartment. DivIVA has a high affinity for negative, i.e., concave, membrane curvature, which is present where the septum meets the lateral edge of the cell (*31*). We note that the forespore side of the septum appears to present higher concavity than the mother cell side due to the rounded shape of the forespore, which could promote DivIVA assembly. We also note that divisome components begin septum synthesis at a second short-lived division site (*32*) at the opposite end of the mother cell. Thus, DivIVA in ring_M_ might redistribute there upon the synthesis of the partial septum, which is eventually abolished (*13*). Since the invaginating septum is thinner in sporulating than vegetative cells, we expected shorter distances between DivIVA dual rings in sporulating cells. We determined the distance between DivIVA rings was similar in vegetative and sporulating cells (Fig. 1J). Our results suggest differences in forespore compartment membrane curvature or additional protein factors might influence DivIVA dual ring localization in sporulating cells. Lastly, we determined that the Z-ring localizes closer (~25 nm) to DivIVA ring_M_ (Fig. 2) and, by extension, the Min system, which disassembles the divisome after division (*33*). We predict that closer proximity to Min proteins enables faster divisome disassembly for the relocalization of FtsZ to the second division site.

We found that the ring assemblies of SepF and FtsZ rings contain different protein amounts between division modes. SepF rings in sporulating cells contained approximately 30% less protein compared to vegetative cells (Fig. 3). SepF, EzrA, and FtsA bind the same 20–amino acid FtsZ protein domain (*8*, *34*, *35*). Thus, there might be competition for Z-ring binding between early divisome proteins. Furthermore, in sporulation, additional proteins localize to the division plane for processes like DNA translation or septal thinning (*12*) and compete for space at the septum. It was proposed that the number of FtsZ filaments at the division site correlates with septum thickness in dividing cells (*12*). We observed that Z-rings in sporulating cells contained about 10% less FtsZ compared to vegetative cells (Fig. 3). However, the division septum is 50% thinner in sporulating cells. Our results suggest that the amount of FtsZ filaments at the constricting septum is not a key determinant of septal thickness. Previous work compared FtsZ protofilament length at active division sites via cryo-focused ion beam cryo-electron tomography (cryo-FIB-ET) slices (*12*). In contrast, our analysis pipeline normalized protein density in rings for Z-ring diameter and analyzed the entire 3D Z-ring assembly with improved statistics.

The nanometer-scale resolution in combination with the quantitative nature of DNA-PAINT allowed us to reveal differences and similarities between the organization and distribution of division proteins in vegetative and sporulating *B. subtilis*. It is very likely that additional divisome components distribute, organize, and assemble differently between modes of division and certainly across species. It will be interesting to see whether the asymmetric placement of divisome proteins is specific to sporulating *B. subtilis* or a universal mechanism across spore-forming bacteria. The ability to simultaneously image and quantify divisome proteins in hundreds of bacteria at nanoscale resolution with high labeling and detection efficiency in situ opens up avenues for mapping the nanoscale structure of the complete divisome and other multicomponent bacterial complexes in the future.

## MATERIALS AND METHODS

### Bacterial culture conditions

Competent *B. subtilis* was created as previously described (*36*). Strains were plated on LB agar plates and grown overnight in LB at 30°C. Sporulating cells and samples for microscopy were prepared as described previously (*37*). Briefly, cells were grown in diluted LB media (1:4) to OD_600_ (optical density at 600 nm) ~ 0.5 to 0.7 and then induced to sporulate using Sterlini and Mandelstam medium at 37°C. Sporangia were fixed at time points between 1.75 and 2.6 hours. Vegetative cells were diluted 1:1000 in SMG media and grown at 30°C until OD_600_ ~ 0.15 then fixed.

### Strain construction

See *B. subtilis* strains used in this study in table S1. *B. subtilis* 168 was used as the background for all strain construction. Primer sequences are found in table S3. A five-part Gibson assembly (*38*) was performed to create plasmids pKCB300 and pKCB1113. pKCB300 contains genes coding for sfGFP and a spectinomycin resistance marker flanked by the DivIVA gene and its downstream region for site-directed homologous recombination. pKCB1113 contains genes coding for sfGFP and a kanamycin resistance marker flanked by the SepF gene and its downstream region for site-directed homologous recombination. A linker was inserted before sfGFP and ALFA-tag by adding codons codifying for 3× glycine to the forward primer used for amplification. After Gibson assembly, plasmids were transformed into competent *B. subtilis* 168 and colony-purified. gDNA was sequenced to verify correctness. pKCB328 was created via a blunt-end cloning kit (NEB, catalog no. E1202S) of a custom gene block (IDT Technologies) (table S6). Strains and plasmids are available upon request.

#### Buffers

The following buffers were used for sample preparation and imaging:

1) PBSG: 1× phosphate-buffered saline (PBS; pH 7.4) and 20 nM glucose

2) PBST: 1× PBS (pH 7.4) and 0.02% (w/v) Tween 20

3) SDS buffer: 1× PBS (pH 7.4) and 0.01% (w/v) SDS

4) Blocking buffer: 3% (w/v) bovine serum albumin, 0.02% (v/v) Tween 20, and 1% (w/v) dextran sulfate (Sigma-Aldrich, catalog no. D4911-10G) in 1× PBS (pH 7.4)

5) Buffer C: 500 mM NaCl in 1× PBS (pH 7.4)

6) SMG media: 15 mM (NH_4_)_2_SO_4_, 61 mM K_2_HPO_4_, 44 mM KH_2_PO_4_, 3.4 mM sodium citrate 2× H_2_O, 1.7 mM MgSO_4_, 5.9 mM glutamate, and 27 mM glucose supplemented with 1.0 mM tryptophan

### DNA-PAINT sample preparation vegetative cells

Cells were fixed, immobilized, permeabilized, and blocked as previously described (*39*). Protein targets were labeled as described below (DNA-PAINT immunolabeling). Cells were washed three times with PBST. Gold nanoparticles (Cytodiagnostics, catalog no. G-90-100) were diluted 1:4 in PBS and added to the sample for 5 min. Cells were then washed three times with PBS. Imager strands were diluted into buffer C containing the PCA (3,4-dihydroxybenzoic acid), PCD (protocatechuate 3,4-dioxygenase pseudomonas), and Trolox oxygen scavenging system and added to samples directly before DNA-PAINT imaging. If vegetative and sporulating cells were to be imaged together, then they were fixed and permeabilized separately before cell immobilization and immunolabeling in the same sample.

### DNA-PAINT sample preparation of sporulating cells

Cells were fixed and immobilized as previously described (*39*). The bacterial cell wall was permeabilized by incubating samples with lysozyme (2 mg/ml; Thermo Fisher Scientific, catalog no. 90082) for 60 s at 30°C. Cells were then washed with SDS buffer three times. Next, a blocking solution was added for 20 min at room temperature (RT). Immunolabeling was performed as described below (DNA-PAINT immunolabeling).

### DNA-PAINT immunolabeling

#### DivIVA labeling

Anti-GFP nanobody (Nano-Tag, catalog no. N0305-250 μg) conjugated with a DNA-PAINT handle was added in a 1:200 dilution and incubated at 4°C overnight (ON).

#### ALFA-tag labeling

Anti-ALFA nanobody (Nano-Tag, catalog no. N1505-250 μg) conjugated with a DNA-PAINT handle was added in a 1:200 dilution and incubated at 4°C ON.

#### FtsZ labeling

Primary rabbit anti-FtsZ antibody (Biozol, catalog no. GTX36253) was added in a 1:100 dilution and incubated at 4°C ON. A secondary anti-rabbit nanobody conjugated with a DNA-PAINT handle was added in a 1:200 dilution and incubated for 90 min at RT.

#### Multiplexed labeling

Binders were added as explained above and incubated ON at 4°C. If imaging FtsZ, the secondary anti-rabbit nanobody conjugated with a DNA-PAINT handle was added the next day in a 1:200 dilution and incubated for 90 min at RT.

Cells were washed three times with PBST. Gold nanoparticles (Cytodiagnostics, catalog no. G-90-100) were diluted 1:4 in PBS and added to the sample for 5 min. Cells were washed three times with PBSG. Imager strands were diluted into buffer C containing PCA, PCD, and Trolox oxygen scavenging system and added to samples directly before DNA-PAINT imaging. Exchange-PAINT was performed by washing samples three times with PBS between imaging rounds, or until no blinking was observed.

### Nile Red imaging

Nile Red (Invitrogen, catalog no. N1142) was added to the sample after all DNA-PAINT imaging was completed. Nile Red was diluted to a concentration of 1 to 1.5 nM in buffer C containing the PCA, PCD, and Trolox oxygen scavenging system and incubated for 5 min before imaging. Once Nile Red was added, no more DNA-PAINT imaging was performed in the sample.

### Preparation of PCA, PCD, and Trolox

40× PCA: 154 mg PCA (Sigma-Aldrich, catalog no. 37580-25G-F) was dissolved in 10 ml of water and adjusted to pH 9.0 using NaOH (Merck, catalog no. 1091361000). 100× PCD: 9.3 mg PCD (Sigma-Aldrich, catalog no. P8279) was dissolved in 13.3 ml of buffer [100 mM tris-HCl (pH 8), 50 mM KCl (Merck, catalog no. 7647-14-5), 1 mM EDTA, and 50% glycerol (Sigma-Aldrich, catalog no. 65516-500ml)]. 100X Trolox: 100 mg (±)-6-hydroxy-2,5,7,8-tetramethylchromane-2-carboxylic acid (Trolox, Sigma-Aldrich, catalog no. 238813-5G) was dissolved in 3.2 ml of H_2_O complemented with 430 μl 100% methanol (Sigma-Aldrich, catalog no. 32213-2.5L) and 345 μl of 1 M NaOH.

### DNA-binder conjugation

The nanobody binder FluoTag-Q anti-GFP (NanoTag, catalog no. N0301) was used to target sfGFP. Nanobody anti-ALFA nanobody (NanoTag, catalog no. N1505) was used to target ALFA-tag. A custom-made anti-rabbit nanobody was used to target primary FtsZ antibodies. All nanobodies contained a single cysteine that was coupled with DNA oligonucleotides functionalized with an azide group at the 5′-end (Metabion, Planegg, Germany), as described previously (*40*).

### Affinity purification of anti-FtsZ antibody

FtsZ was purified on the basis of an affinity purification method by following the protocol from the manufacturer https://tinyurl.com/y5zutwhd. Since FtsZ antibodies (Biozol, catalog no. GTX36253) were only available in small volumes (microliters), bead coupling and purification were performed in 1.5-ml Eppendorf tubes. Thus, washing is defined as centrifugation and removal of supernatant. Elution fractions were dialyzed using the Slide-A-Lyzer Mini Dialysis device (Thermo Fisher Scientific, catalog no. 69550) into PBS (w/v) 50% glycerol for storage at −20°C.

### DNA-PAINT microscope setup

DNA-PAINT imaging was carried out on an inverted microscope (Nikon Instruments, Eclipse Ti2) with the Perfect Focus System, applying an objective-type total internal reflection fluorescence (TIRF) configuration equipped with an oil-immersion objective (Nikon Instruments, Apo SR TIRF 100×, NA 1.49, oil). A 488-nm (200 mW, Toptica iBeam smart) or 561-nm laser (Coherent Sapphire, 200 mW) was used for excitation and was coupled into a single-mode fiber. The laser beam was passed through cleanup filters (Chroma Technology, ZET561/10) and coupled into the microscope objective using a beam splitter (Chroma Technology, ZT561rdc). Fluorescence light was spectrally filtered with an emission filter (Chroma Technology, ET600/50 m) and imaged with a scientific complementary metal-oxide semiconductor camera (Andor, Zyla 4.2plus) without further magnification, resulting in an effective pixel size of 130 nm after 2 × 2 binning. The camera readout sensitivity was set to 16-bit, and readout bandwidth to 540 MHz. Three-dimensional imaging was performed using a cylindrical lens (Nikon Instruments, N-STORM) in the detection path.

### Image analysis

Raw fluorescence data from DNA-PAINT imaging was subjected to super-resolution reconstruction using the “Picasso” software package (*18*) (latest version available on https://github.com/jungmannlab/picasso). Drift correction was performed with a redundant cross-correlation and gold nanoparticles as fiducials. Gold nanoparticles were also used to align the FtsZ and DivIVA signals, FtsZ and SepF signals, and FtsZ and ZapA signals in Exchange-PAINT. Regions of interest (whole cells or septa) were selected manually using Picasso’s “Pick tool” and the rectangle pick option. Cells and septa were picked along the main axis of the bacterial cell, in sporulating cells in the direction from the forespore ring to the mother ring, yielding rotated *xy* coordinates. Statistical analyses were performed and graphs were created using the software Origin 2019b from OriginLab. The notation of *P* values is as follows: * indicates 1.00 × 10^−02^ < *P* ≤ 5.00 × 10^−02^. ** indicates 1.00 × 10^−03^ < *P* ≤ 1.00 × 10^−02^. *** indicates 1.00 × 10^−04^ < *P* ≤ 1.00 × 10^−03^. **** indicated *P* ≤ 1.00 × 10^−04^. IQR refers to the second and third data quartiles (middle 50% of data). Localization precisions were calculated using a nearest neighbor–based analysis (*41*) and are listed in table S8. The mean localization precision for all DNA-PAINT measurements was 6 ± 1 nm (means ± SD, *n* = 414).

### Ring analysis workflow

First, the longitudinal position of the Z-ring in sporulating cells was determined by a unimodal Gauss fit in a histogram (bin size, 20 nm) representing frequency counts of localizations along the main axis of each picked cell. The location of the cell within the picked ROI was identified using the signal from cytosolic FtsZ: In the histogram, a bin was considered to show the signal if it contained more than 10% of the median number of counts in nonzero bins. Identifying the first and last block of 10 consecutive bins above this threshold allows us to identify the cell edges as the outermost bins of these blocks. In this way, noise at the edges of the picked ROI will not induce artifacts in the identification of the longitudinal position of the cell within the region of interest. Subsequently, the relative position of the Z-ring within the cell was calculated.

First, individual rings were identified using frequency counts along the rotated *y* axis of every picked septum. The distributions were fitted using unimodal (FtsZ, SepF, and ZapA) or bimodal (DivIVA) Gaussian distributions. The fits were initialized using the height and width estimations obtained from the mean shift algorithm (*42*), which is implemented in the Python package scikit-learn (*43*). For dual-band data, the identified rings were mutually filtered using the values for width, amplitude, and distance to exclude picks where the two bands could not be successfully identified. Distances between DivIVA rings (Fig. 1, I and L), between DivIVA and FtsZ rings (Fig 2, E and J), between FtsZ and SepF, and between FtsZ and ZapA (Fig. 3, E and J, and fig. S4) were calculated from the center-to-center distance of the respective Gauss peaks.

Localizations from individual rings were selected in a corridor around the center of the Gaussian fit of the frequency count. Here, the corridor width is scaled with the width of the Gaussian fit (fig. S6).

Selected localizations were further used to perform a tilt correction of single-band data and individual DivIVA rings: Therefore, a singular value decomposition (SVD) was used to estimate the transformation matrix. Subsequently, Rodrigues’ rotations were applied to the original set of localizations resulting in a corrected alignment of the ring plane with the *xz* plane. Recalculating the frequency count and the unimodal Gauss fit yields a narrower curve width, thus allowing for a more precise selection of ring localizations in a corridor around the center of the Gauss fit.

Next, the rings were fitted using a circle fit in the *xz* plane. The circle fits revealed ring radii and ring center information. For single-band data, only localizations with *xz* distances to the ring center being within ±100 nm of the fitted ring radius were kept for further analysis. If less than 80% of the localizations are within this region, then the ring was discarded. Furthermore, a ring was excluded if it was not sampled all around: To this aim, 12 segments were defined along the ring. A segment is considered to be sampled if it contains at least a fourth of the localizations that it would contain if the localizations *N*_locs_ were evenly distributed [*N*_locs_/(4*N*_segments_)]. If this criterium is fulfilled by less than eight segments, then the ring is discarded.

For the qPAINT analysis, selected localizations were linked, and kinetic information including the dark time τ_d_ was determined for every ring using the algorithm described earlier (*18*, *19*).

To identify whether one of the DivIVA rings contained more protein copies than the other, the ratio of the dark time of the forespore ring and the mother ring (sporulating cells) or ring 1 and ring 2 (vegetative cells) was calculated. A value larger than 1 indicates a higher protein amount in the forespore ring than in the mother ring or in ring 1 compared to ring 2. These ratios were calculated for all cells in all imaged FOVs and lastly compared between vegetative and sporulating cells.

To identify whether sporulating or vegetative cells contain more protein in their respective FtsZ, SepF, and ZapA rings, ring radii need to be considered because the protein amount in a ring scales with the radius.

In addition, protein amounts of sporulating and vegetative cells can only be compared within the same FOV because the dark times τ_d_ become incomparable if experimental conditions minimally differ between experiments. Therefore, for each FOV, the dark times are first plotted against their respective ring radius *r*. Then, a linear fit was performed for sporulating and vegetative data points, respectively: 1/τ_d_ = *a* * *r*, where *a* is the slope of the fitted line. A larger slope indicates a higher protein density across the ring. The ratio of these slopes (*a*_spor_/*a*_veg_) can be compared across FOVs. The statistical significance of the calculated mean ratios displayed in Fig. 3M was first determined with a one-sampled *t* test, which found that SepF and FtsZ means are significantly different from a ratio of 1 (SepF: *P* = 4.49736 × 10^−4^; FtsZ: *P* = 2.05054 × 10^−5^), but not ZapA (*P* = 0.56196). In addition, a two-sampled *t* test (Welch’s test) determined that the Z-ring and SepF have statistically significant distributions (*P* = 0.00456), while ZapA and SepF (*P* = 0.20666) as well as ZapA and FtsZ (*P* = 0.90987) do not. SVD and circular fitting were performed using the SciPy package (*44*) (v.1.4.1).

### SepF arc diameter

First, the tip of SepF arcs was manually selected using Picasso’s Pick tool. Second, a *z* slice of 100-nm thickness is selected by fitting a unimodal Gaussian to a histogram along the *z* axis of the pick. Only localizations within ±50 nm around the Gauss peak are considered further. Last, a histogram along the *x* axis of the rectangle pick is calculated and SepF arc tips are identified by a bimodal Gauss fit. Using the values for width, amplitude, and distance, picks are discarded if the two peaks cannot be successfully identified. The arc diameter is the distance between both Gauss peaks.

## Supplementary Materials

### This PDF file includes:

Figs. S1 to S7 Tables S1 to S8 References
