# Supplementary material for: Comparing divisome organization between vegetative and sporulating Bacillus subtilis at the nanoscale using DNA-PAINT
Source: Sci Adv. 2024 Jan 10;10(2):eadk5847. doi: 10.1126/sciadv.adk5847 (PMC10780868; doi:10.1126/sciadv.adk5847)
Supplement: Supplementary file 1 — Figs. S1 to S7 Tables S1 to S8 References [file sciadv.adk5847_sm.pdf]

Science Advances
AAAS

Supplementary Materials for

Comparing divisome organization between vegetative and sporulating *Bacillus subtilis* at the nanoscale using DNA-PAINT

Kimberly Cramer *et al.*

Corresponding author: Jae Yen Shin, shinjaeyen@gmail.com; Ralf Jungmann, jungmann@biochem.mpg.de

Sci. Adv. **10**, eadk5847 (2024)
DOI: 10.1126/sciadv.adk5847

This PDF file includes:

Figs. S1 to S7
Tables S1 to S8
References

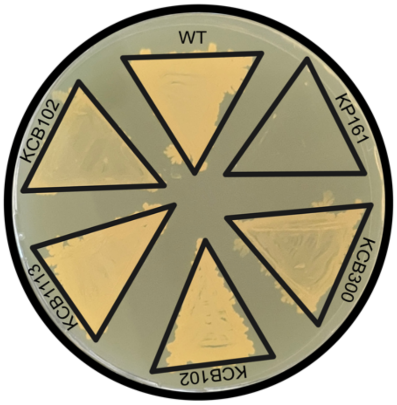

Supplementary Figure 1 | Sporulation assay of strains. A sporulation assay was performed on strains KCB300, KCB1113, KCB328, and KCB102. WT (168) strain was used as a positive control and KP161 used as a negative control. Sporulation positive strains become opaque after nutrient depletion (see WT), indicating spore formation. Sporulation negative strains become transparent, indicating cell death (see KP161). Strains KCB300, KCB1113, KCB328, and KCB102 resemble the WT strain, indicating successful sporulation.

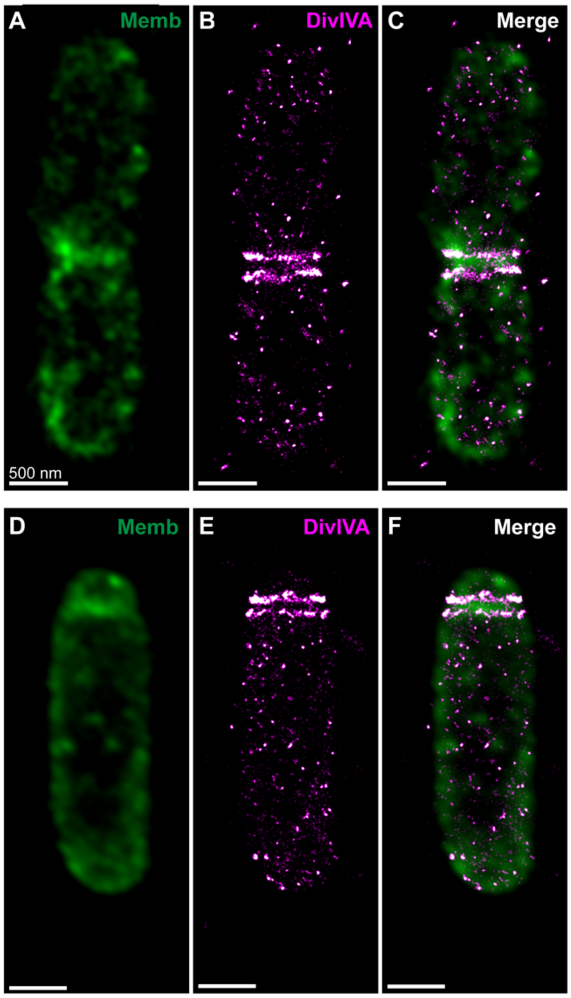

Supplementary Figure 2 | Dual DivIVA rings flank the division septum in vegetative and sporulating *B. subtilis*. DNA-PAINT was performed on DivIVA and PAINT imaging on the cell membrane. DivIVA imaging implemented in strain KCB300 via anti-GFP nanobodies conjugated with a DNA-PAINT handle. Membrane imaging performed using Nile Red PAINT dye. (**A–C**) Imaging results in a vegetative cell. (**A**) Nile Red imaging of the cell membrane in a vegetative cell shows the division septum as a band at mid-cell. (**B**) DNA-PAINT imaging reveals DivIVA localizes as one band on each side of the division septum. (**C**) Merge image of **A** and **B**. (**D–F**) Respective results for a sporulating cell.

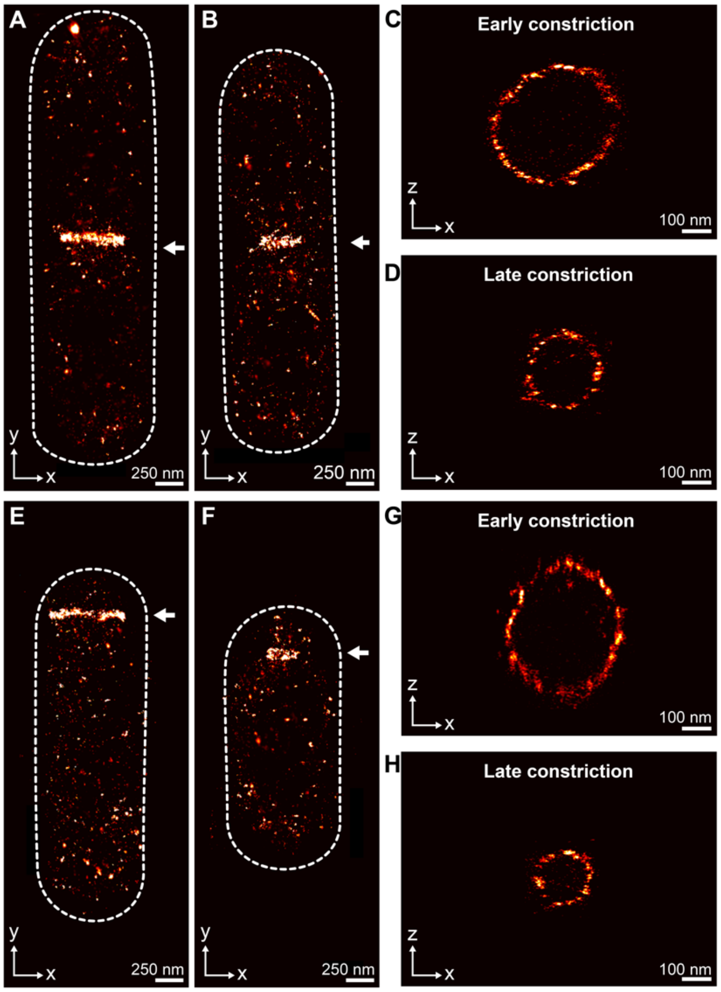

Supplementary Figure 3 | DNA-PAINT imaging of FtsZ in vegetative and sporulating *B. subtilis*. FtsZ imaging was implemented in strain KCB300 via anti-FtsZ primary antibodies and secondary nanobodies conjugated with a DNA-PAINT handle. (**A–D**) FtsZ localization in two vegetative cells. (**A**) DNA-PAINT imaging of FtsZ protein shows localization as a single band at the mid-cell, as expected. (**B**) DNA-PAINT imaging of FtsZ shows a "shorter" FtsZ band at the division septum when compared to **A**. (**C–D**) The zx-projections of the FtsZ bands in a and b are displayed in c and d, respectively. We show that both bands are indeed rings, and that the Z-ring displayed in **A** was fixed at an earlier stage of constriction compared to that of **B***.* White arrows point to FtsZ bands. (**E–H**) Same as in **A–D** but for two sporulating cells. We visualize the Z-ring placed near a single cell pole, as expected.

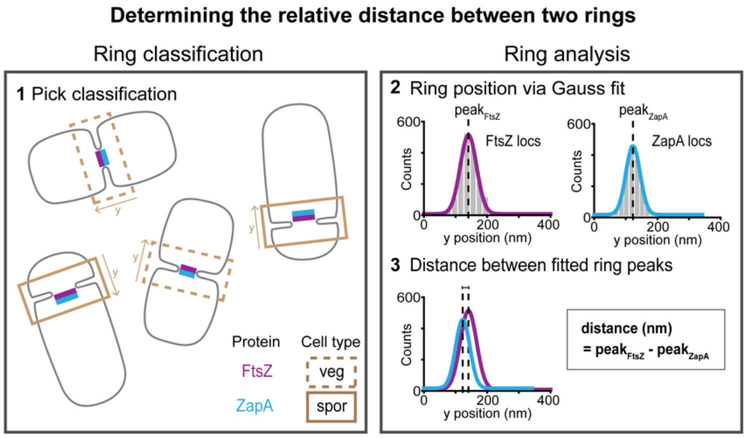

Supplementary Figure 4 | DNA-PAINT data analysis pipeline overview for determining the relative distance between two ring-shaped proteins in *B. subtilis.* (**1**) Rings are first classified via interactive "picking" in Picasso and saved with the corresponding cell type and protein classifier. In sporulating bacteria, the y-axis points towards the mother compartment, while no directionality exists for vegetative cells. (**2**) For each selected region, localizations are binned along the y-axis in the FtsZ and ZapA channel respectively. The positions of the FtsZ and ZapA rings are determined via Gaussian fits. (**3**) The peak position of ZapA was subtracted from the ring position of FtsZ to yield their distance. At sporulation septa, negative distance values indicate that the ZapA ring is closer to the mother cell compartment than the Z-ring and positive values indicate that ZapA is closer to the forespore than FtsZ. Analysis for FtsZ and SepF Exchange-PAINT data was performed analogously.

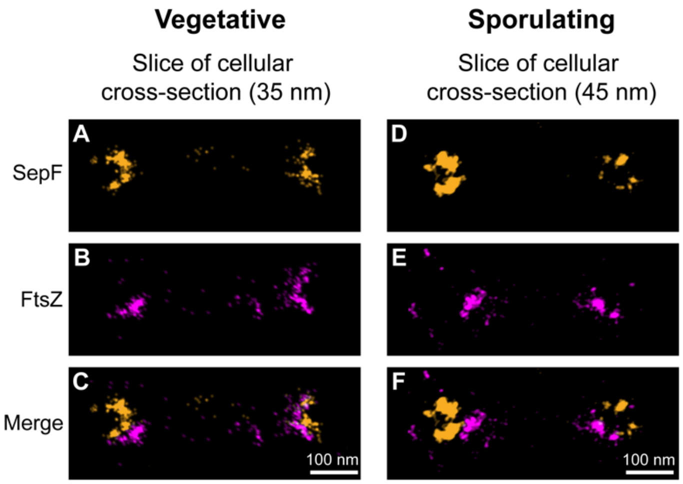

Supplementary Figure 5 | Single slice of 3D DNA-PAINT imaging of SepF and FtsZ in vegetative and sporulating *B. subtilis,* at the cellular cross-section. Imaging performed using strain KCB1113, SepF-sfGFP. FtsZ imaging performed via anti-FtsZ primary antibodies and secondary nanobodies conjugated with a DNA-PAINT handle. SepF imaging performed with anti-GFP nanobodies conjugated with a DNA-PAINT handle. (**A–C**) 35 nm slice of the division plane at the cellular cross-section in a vegetative cell. DNA-PAINT reveals (**A**) SepF assembles as arc shaped structures with endpoints facing away from the bacterial cytosol and (**B**) FtsZ as puncta or an arc shape at the cross-section of the division plane. (**C**) Merge image of **A** and **B** indicate FtsZ assemblies border or localize near to SepF arcs. (**D–F**) 45 nm slice of the division plane at the cellular cross-section in a sporulating cell. (**D**) Again, DNA-PAINT visualizes SepF as two arc shapes with endpoints facing away from the bacterial cytoplasm. (**E**) DNA-PAINT of FtsZ shows FtsZ assembly as puncta. (**F**) Merge image of d and e indicates FtsZ assemblies localize near to or adjoin SepF arcs. SepF in orange. FtsZ in magenta.

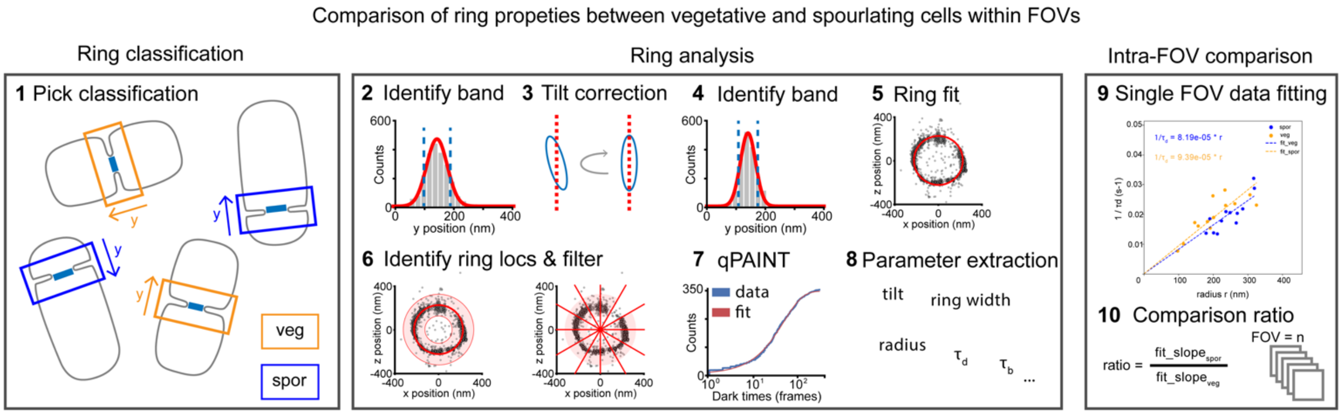

Supplementary Figure 6 | Comparison of ring properties between vegetative and sporulating cells. (**1**) Rings are first classified via interactive "picking" in Picasso and saved with the corresponding cell type classifier. (**2**) A histogram of localizations from an individual pick is calculated. The ring band is identified via a Gaussian fit and ring localizations are selected within a corridor around the peak position. The width of the corridor scales with the width of the Gauss fit. (**3**) Selected localizations are used to measure the tilt via singular value decomposition. The tilt is corrected for all localization in the pick via Rodrigues rotations. (**4**) Repeating the ring band identification on the tilt corrected data results in a narrower width of the Gauss fit and a more precise selection of ring localizations. (**5**) A circle is fitted to the selected ring localizations via least squares fit revealing the ring radius. (**6**) To reduce bias in the quantification of proteins amounts only localizations within ± 100 nm around the fitted ring radius (red shaded area, left panel) will be used. In addition, rings will be discarded if more than 20 % of localizations are outside of this ring region. Moreover, rings are further excluded if they are incompletely sampled along the ring circumference. To this aim the ring was split into 12 segments. A segment is considered to be sampled if it contains at least $N_{locs}/(4N_{segments})$ localizations. If at least 8 segments are sampled the ring is kept for further processing. (**7**) qPAINT was performed by fitting the cumulative distribution function of dark times yielding the mean dark time τ_d. (**8**) The determined ring parameters are saved for every selected ring in the FOV. (**9**) Protein amounts scale with $1/\tau_d$. In order to compare protein amounts the scaling of the inverse dark time with the ring radius was determined via a linear fit to all vegetative and sporulating rings, respectively, occurring in a single FOV. The slope of the linear fit represents protein densities. (**10**) The dark time as a metric for protein density varies with experimental conditions. To ensure comparability across FOVs the ratio between the sporulating and vegetative fit slopes is calculated, which measures the difference in protein amounts between both cell types.

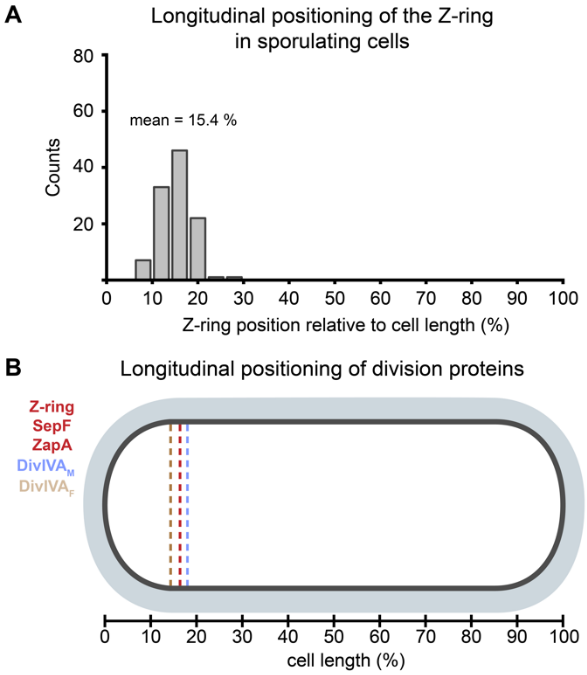

Supplementary Figure 7 | Longitudinal positioning of the Z-ring, SepF, ZapA, and DivIVA in sporulating cells. (**A**) Distribution of the longitudinal position of the Z-ring relative to cell length in individual sporulating *Bacillus subtilis* (KCB300). The Z-ring is typically positioned at 15 % ± 4 % (mean ± std, n = 110) of the cell length. (**B**) Schematic representation of the positioning of division proteins relative to cell length. Pictured cell is 2.8 μm long. SepF and ZapA rings are positioned with the Z-ring at approx. 15 % of the cell length (431 nm), indicated by red dotted line. Positioning of SepF and ZapA derived from results in **Fig. 3** of the Main text. Using the previously calculated mean distances of the Z-ring from rings DivIVA$_F$ and DivIVA$_M$ (see **Fig. 2**), DivIVA$_F$ and DivIVA$_M$ are positioned at approx. 13 % (360 nm) ± 1 % and 17 % (479 nm) ± 1 % of the cell length, respectively (mean ± std). Brown dotted line indicates positioning of DivIVA$_F$. Blue dotted line indicates mean positioning of DivIVA$_M$. DivIVA$_F$ refers to the DivIVA ring in the forespore compartment. DivIVA$_M$ refers to the DivIVA ring in the mother cell compartment. Cell membrane in black. Cell wall in grey. Cytoplasm in white. Cell length (%) refers to length of the cell body, excluding the cell wall.

Supplementary Table 1 | Strain information

Name	Genotype/Description	Reference or construction
168	Wild type (WT)	Burkholder et al(*45*)
KP161	*spoIIGB::erm*	Kenney et al(*46*)
KCB300	*divIVA::divIVA-GlyGlyGly-sfGFP spec*	pKCB300 → 168
KCB1113	*sepF::sepF- GlyGlyGly-sfGFP kan*	pKCB1113 → 168
KCB328	*zapA::zapA-ALFA kan*	pKCB328 → 168
KCB102	*zapA::zapA-ALFA kan*<br>*divIVA::divIVA-GlyGlyGly-sfGFP spec*	gDNA KCB328 → 168

Supplementary Table 2 | Plasmid information

Name	Insert Description	Construction
pKCB300	divIVA-GlyGlyGly-sfGFP spec downstream-divIVA	Five-part Gibson Assembly
pKCB1113	sepF-GlyGlyGly-sfGFP kan downstream-sepF	Five-part Gibson Assembly
pKCB328	zapA-ALFA kan downstream-zapA	Gene block insertion into pMINI2.0 vector (NEB)

Supplementary Table 3 | Primer sequences

Name	Sequence (5' → 3')
JS029	AATTGGATGATATTTAGCGTATTTTGGAAAAGTTAATCG
JS030	GAATATCATTTGGCGTTAATGGCATGATCCCCGGGTACCGAGC
JS031	ATGCCATTAACGCCAAATG
KC050	CTTTGCTCATTCCTTTTCCTCAAATACAGC
KC051	AGGAAAAGGAGGAGGAGGAATGAGCAAAGGAGAAGAAC
KC052	ATTATACAGATTATTTGTAGAGCTCATCC
KC053	CTACAAATAATCTGTATAATAAAGAATAATTATTAATCTGTAG
JS036	TCAGAGAATTTATTAATTGAGAGAAGTTTCTATAGAATTTTTC
JS037	TTCTCTCAATTAATAAATTCTCTGATTATCTTGACATTTTC
11131F	ACAGGAGGCAGTAATTGGATGATATTTAGCGTATTTTGGAAAAGTTAATCGCCG
11131R	CCGCTCTGTCTCATGGATCCCCGGGTACCGAGC
11132F	CCCGGGGATCCATGAGACAGAGCGGGAATCTC
11132R	TCATACCGCCACCCCACCTCTGATGTTCGTCTTC
11133F	CATCAGAGGTGGGGTGGCGGTATGAGCAAAGG
11133R	AATACCAGAAAATTATTTGTAGAGCTCATCCATGCC
11134F	GCTCTACAAATAATTTTCTGGTATTTAAGGTTTTAG
11134R	TTGTGCCTTTGCCTAAAACAATTCATCCAGTAAAATATAATATTTTATTTTC
11135F	TGAATTGTTTTAGGCAAAGGCACAATATCAGCTTG
11135R	ATATCATCCAATTACTGCCTCCTGTGCTCTTTC
IKCB328A	CTGTACGTCATTTTCATTTGG
IKCB238B	CATCATTTGCTGCAACG

Supplementary Table S4| Imager and docking strand sequences

Name	Sequence (5' → 3')	Modifications	Vendor
R1 docking	TTTCCTCCT	5' C-3 azide	Metabion
R3 docking	TCCTCTCTC	5' C-3 azide	Metabion
5xR1 docking	TCCTCCTCCTCCTCCT	5' C-3 azide	Metabion
5xR2 docking	ACCACCACCACCACCA	5' C-3 azide	Metabion
7xR3 docking	CTCTCTCTCTCTCTCTC	5' C-3 azide	Metabion
7xR4 docking	ACACACACACACACACA	5' C-3 azide	Metabion
R1 imager	AGGAGGA	3'-Cy3B	Metabion
R2 imager	TGGTGGT	3'-Cy3B	Metabion
R3 imager	GAGAGAG	3'-Cy3B	Metabion
R4 imager	TGTGTGT	3'-Cy3B	Metabion

Supplementary Table S5 | DNA-PAINT imaging parameters

Target	Integration time	Number of frames	Imager concentration	Laser power at objective
DivIVA	100 - 200 ms	10,000 – 20,000	100 - 500 pM	20 - 25 mW
FtsZ	100 - 200 ms	10,000 – 20,000	75 - 300 pM	20 - 25 mW
ZapA	100 - 200 ms	10,000 – 20,000 per target	100 - 500 pM	20 – 25 mW
SepF	100 - 125 ms	10,000 – 20,000	100 - 500 pM	20 – 25 mW

Supplementary Table S6 | PAINT imaging parameters

Target	Integration time	Number of frames	Nile Red concentration	Laser power at objective	Localization precision
Membrane	40 ms	40,000	1.5 nM	45 mW	17.1 nm

Supplementary Table S7 | DNA sequence of gene block used to make pKCB328

Gene block sequence
CTGTACGTCATTTTCATTTGGTCAATAGCAGACAACGATACTTTTATCACTGAATGGGACACGTAATAATCTCC<br>TTTTTTTACACTTTTCGCTGTATATACCAGTGTATCATAACAGCGGGAGGCTCGTCTTTCCATTCATTTAATAA<br>ACGTGTTATGATAAGAACTAGGATTCTCGCGGAATGGAGGAGAAACGTTGTCTGACGGCAAAAAAACAAAAACA<br>ACCGTTGACATTTACGGCCAGCACTTCACGATTGTCGGTGAAGAAAGCAGAGCCCATATGAGGTATGTCGCCGG<br>AATTGTTGATGATAAAATGAGAGAAATCAATGAAAAAAATCCATACCTTGATATAAATAAACTTGCAGTGCTGA<br>CAGCGGTAAATGTGGTGCACGATTATGTCAAATTACAAGAGAAATGTGAAAAACTGGAGCGTCAGCTTAAAGAA<br>AAGGATCCTAGCCGTTTGGAAGAGGAACTGAGACGCCGTTTAACTGAATAATACCGTTCGTATAGCATACATTA<br>TACGAAGTTATTTTTCTGGTATTTAAGGTTTTAGAATGCAAGGAACAGTGAATTGGAGTTCGTCTTGTTATAAT<br>TAGCTTCTTGGGGTATCTTTAAATACTGTAGAAAAGAGGAAGGAAATAATAAATGGCTAAAATGAGAATATCAC<br>CGGAATTGAAAAAACTGATCGAAAAATACCGCTGCGTAAAAGATACGGAAGGAATGTCTCCTGCTAAGGTATAT<br>AAGCTGGTGGGAGAAAATGAAAACCTATATTTAAAAATGACGGACAGCCGGTATAAAGGACCACCTATGATGT<br>GGAACGGGAAAAGGACATGATGCTATGGCTGGAAGGAAAGCTGCCTGTTCCAAAGGTCCTGCACTTTGAACGGC<br>ATGATGGCTGGAGCAATCTGCTCATGAGTGAGGCCGATGGCGTCCTTTGCTCGGAAGAGTATGAAGATGAACAA<br>AGCCCTGAAAAGATTATCGAGCTGTATGCGGAGTGCATCAGGCTCTTTCACTCCATCGACATATCGGATTGTCC

CTATACGAATAGCTTAGACAGCCGCTTAGCCGAATTGGATTACTTACTGAATAACGATCTGGCCGATGTGGATT<br>GCGAAAACTGGGAAGAAGACACTCCATTTAAAGATCCGCGCGAGCTGTATGATTTTTTAAAGACGGAAAAGCCC<br>GAAGAGGAACTTGTCTTTTCCCACGGCGACCTGGGAGACAGCAACATCTTTGTGAAAGATGGCAAAGTAAGTGG<br>CTTTATTGATCTTGGGAGAAGCGGCAGGGCGGACAAGTGGTATGACATTGCCTTCTGCGTCCGGTCGATCAGGG<br>AGGATATCGGGGAAGAACAGTATGTCGAGCTATTTTTTGACTTACTGGGGATCAAGCCTGATTGGGAGAAAATA<br>AAATATTATATTTTACTGGATGAATTGTTTTAGACAACTATGCTAGATATCATCATCTTAATCTTGCTCCTGAT<br>GGGGACTTTACTGGGGTTAAAACGCGGTTTTATCCTGCAGTTTATCCGCTTGACGAGCTTTATTTTATCAATTG<br>CCTTTGCGGCTTTATTCTATAAAAACGTGGCACCGCATTTACATTGGATTCCCGCACCCGATTTTTCAGCGGGA<br>CAGCCGGCTCTTTCTTTTTTTACGGGGAATTTGGAAGCAGCGTACTATAATGCGATTGCGTTTATCGTTTTATT<br>TATCATTGCTAAAATCTTACTGAGAATCATCGGCTCGTTCCTGAGTATTGTAGCCGGCATTCCGGTGATTAAAC<br>AAATCAACCAGATGCTGGGAGCCGTTCTCGGTTTTCTAGAAGTCTATTTATTTACATTTGTGCTGCTGTATGTC<br>GCATCCGTTCTGCCGGTAGACGCGTTGCAGCAAATGATG

Supplementary Table S8 | List of DNA-PAINT imaging datasets and their localization precisions

Dataset	Loc. Precision (nm)
230401_fov4_kcb1113_500pMr4_SepF_DP_1_drift_aligned_picked.hdf5	6.35
230518_artemis_kcb1113_fov4_140pM-r2_SepF_DP_1_drift_aligned_picked.hdf5	4.83
230518_artemis_kcb1113_fov1_150pM-r2_SepF_DP-2_1_drift_aligned_picked.hdf5	4.73
230310_kcb1113_2plex_spor_fov5_500pM-r3_SepF_DP_3_drift_picked.hdf5	6.47
230518_artemis_kcb1113_fov2_150pM-r2_SepF_DP_1_drift_aligned_picked.hdf5	5.22
230401_fov2_kcb1113_50pMr2_SepF_DP_1_drift_filter_sxsy_picked.hdf5	6.14
221015_2plex_rab7xR4_gfp5xR1_kcb1113-fov1-SepF_2_drift_aligned_picked.hdf5	5.27
230310_kcb1113_2plex_spor_fov3_500pM-r3_SepF_DP_1_drift_aligned_picked.hdf5	4.82
230401_fov4_kcb1113_500pMr4_SepF_DP_1_drift_aligned_picked.hdf5	6.28
230310_kcb1113_2plex_spor_fov3_500pM-r3_SepF_DP_1_drift_aligned_picked.hdf5	5.04
230518_artemis_kcb1113_fov2_150pM-r2_SepF_DP_1_drift_aligned_picked.hdf5	4.10
230401_fov1_kcb1113_500pMr4_SepF_DP_1_drift_filter-2_picked.hdf5	7.50
230518_artemis_kcb1113_fov3_150pM-r2_SepF_DP_1_drift_aligned_picked.hdf5	5.03
230401_fov2_kcb1113_50pMr2_SepF_DP_1_drift_filter_sxsy_picked.hdf5	6.74
230310_kcb1113_2plex_spor_fov4_500pM-r3_SepF_DP_1_drift_aligned_picked.hdf5	5.47
230518_artemis_kcb1113_fov4_140pM-r2_SepF_DP_1_drift_aligned_picked.hdf5	4.66
230310_kcb1113_2plex_spor_fov2_500pM-r3_SepF_DP_1_drift_aligned_picked.hdf5	5.56
230401_fov3_kcb1113_500pMr2_SepF_DP_1_drift_aligned_picked.hdf5	6.26
221015_2plex_rab7xR4_gfp5xR1_kcb1113-fov1-SepF_2_drift_aligned_picked.hdf5	7.56
230310_kcb1113_2plex_spor_fov2_500pM-r3_SepF_DP_1_drift_aligned_picked.hdf5	5.37
230518_artemis_kcb1113_fov1_150pM-r2_SepF_DP-2_1_drift_aligned_picked.hdf5	5.28
230310_kcb1113_2plex_spor_fov5_500pM-r3_SepF_DP_3_drift_picked.hdf5	6.77
230401_fov3_kcb1113_500pMr2_SepF_DP_1_drift_aligned_picked.hdf5	5.79
230518_artemis_kcb1113_fov3_150pM-r2_SepF_DP_1_drift_aligned_picked.hdf5	4.62
230310_kcb1113_2plex_spor_fov4_500pM-r3_SepF_DP_1_drift_aligned_picked.hdf5	5.73
230401_fov1_kcb1113_500pMr4_SepF_DP_1_drift_filter-2_picked.hdf5	6.33

230513_Gemini_fov3_Zring_200pM_FtsZ_DP_1_drift_picked.hdf5	5.01
221006_spor_KCB324_rab5xR2_GFP5xR1_fov2_FtsZ_1_aligned_picked.hdf5	6.03
200524_test_0p75_fov0_dp_ftsz_1_drift_picked.hdf5	5.72
221015_2plex_rab7xR4_gfp5xR1_kcb1113-fov3-FtsZ_1_drift_picked.hdf5	5.18
221006_spor_KCB324_rab5xR2_GFP5xR1_fov1_FtsZ_DP_1_aligned_PICKED.hdf5	9.10
200618_exchange1_fov7_ftsz_dp_1_drift_aligned_picked.hdf5	4.90
230314_kcb300_spor_RBnb5xr2_200pm-r2_fov2_FtsZ_DP_1_drift_picked.hdf5	5.31
230401_fov2_kcb1113_150pMr2_FtsZ_DP_1_drift_picked.hdf5	6.94
230513_artemis_Zring_fov3_200pM-r2_ftsz_DP_1_drift_picked.hdf5	5.66
230513_apollo_Zrings_fov1_r2-125pM_FtsZ_DP_1_drift_picked.hdf5	5.70
230513_artemis_Zring_fov8_300pM-r2_ftsz_DP_1_drift_picked.hdf5	5.39
200524_test_0p75_fov0_dp_ftsz_1_drift_picked.hdf5	4.58
230513_Gemini_fov3_Zring_200pM_FtsZ_DP_1_drift_picked.hdf5	5.01
220913_kcb306_spor_alfa2xr3_rab5xR2_fov2_200pM-R2_FtsZ_1_drift_aligned_PICKED.hdf5	5.17
230429_fov3_kcb300_115pM-r2_20mW_ftsz_DP_2_drift_aligned3_picked.hdf5	6.50
230513_Gemini_fov1_Zring_FtsZ_DP_1_drift_picked.hdf5	4.91
230513_artemis_Zring_fov8_300pM-r2_ftsz_DP_1_drift_picked.hdf5	4.94
230317_fov3_2plex_kcb1113_200pM-r2_DP_FtsZ_1_DRIFT_aligned_picked.hdf5	4.96
230309_kcb1113_veg_2plex_fov4_300pM-r2_FtsZ_DP_1_drift_picked.hdf5	5.11
230513_apollo_Zrings_fov7_r2-300pM_FtsZ_DP_1_drift_picked.hdf5	6.35
230513_Gemini_fov10_Zring_300pM_FtsZ_DP_1_drift_aligned_picked.hdf5	5.05
230429_fov1_kcb1113_200pM-r2_FtsZ_DP_1_drift_aligned_picked.hdf5	4.04
200716_exch_new_fov20_dp_ftsz_1_aligned_picked.hdf5	5.19
230513_Gemini_fov4_Zring_200pM_FtsZ_DP_1_MMStack_Pos0.ome_locs_picked.hdf5	5.02
230513_Gemini_fov6_Zring_300pM_FtsZ_DP_1_drift_picked.hdf5	4.77
220913_kcb306_spor_alfa2xr3_rab5xR2_fov1_200pM-R2_FtsZ_1_drift_picked.hdf5	4.71
230513_apollo_Zrings_fov9_r2-300pM_FtsZ_DP_1_drift_picked.hdf5	6.42
220429_kcb102_alfaP3-NBrabbit-7xr4_test_fov1-Ftsz_DP_1_drift_aligned_picked.hdf5	4.15
230513_apollo_Zrings_fov10_r2-300pM_FtsZ_DP_1_drift_picked.hdf5	6.64
220913_kcb306_spor_alfa2xr3_rab5xR2_fov2_200pM-R2_FtsZ_1_drift_aligned_PICKED.hdf5	4.44
230310_kcb1113_2plex_spor_fov5_200pM-r2_FtsZ_DP_1_drift_picked.hdf5	7.99
230314_kcb300_spor_RBnb5xr2_200pm-r2_fov4_FtsZ_DP_1_drift_picked.hdf5	4.69
220913_kcb306_spor_alfa2xr3_rab5xR2_fov1_200pM-R2_FtsZ_1_drift_picked.hdf5	4.82
220805_kcb102_fov2_3plex_nbRAB7xR5_FtsZ_1_aligned_PICKED.hdf5	6.31
200618_exchange1_fov8_ftsz_dp_1_aligned_picked.hdf5	4.37
230429_fov3_kcb1113_225pM-r2_ftsz_DP_1_drift_picked.hdf5	4.83
230513_apollo_Zrings_fov5_r2-300pM_FtsZ_DP_1_drift_picked.hdf5	6.26

230310_kcb1113_2plex_spor_fov2_160pM-r2_FtsZ_DP_1_aligned_picked.hdf5	5.98
220428_kcb102_AB-FtsZ_7xR4_50pM_fov1_DP_1_drift_filter_VEG-picked.hdf5	5.86
230429_fov3_kcb1113_225pM-r2_ftsz_DP_1_drift_picked.hdf5	4.49
230314_kcb300_spor_RBnb5xr2_200pm-r2_fov6_FtsZ_DP_1_drift_picked.hdf5	4.25
191218_ftsZ_ntsecondary_fov2_200pM_r3_23mW561_3dDP_1_1_drift - Copy_FtsZ_picked.hdf5	5.77
230513_artemis_Zring_fov5_200pM-r2_ftsz_DP_1_drift_picked.hdf5	4.44
230513_Gemini_fov6_Zring_300pM_FtsZ_DP_1_drift_picked.hdf5	5.07
230513_artemis_Zring_fov3_200pM-r2_ftsz_DP_1_drift_picked.hdf5	5.25
220913_kcb306_spor_alfa2xr3_rab5xR2_fov3_200pM-R2_FtsZ_2_drift_picked.hdf5	4.79
230401_fov3_kcb1113_112pMr2_FtsZ_DP_1_drift_aligned_picked.hdf5	6.62
221006_spor_KCB324_rab5xR2_GFP5xR1_fov3_FtsZ_1_drift_picked.hdf5	6.57
230314_kcb300_spor_RBnb5xr2_200pm-r2_fov2_FtsZ_DP_1_drift_picked.hdf5	4.87
200717_exch_kcb300_fov4_dp_ftsz_1_drift_align_picked.hdf5	4.34
220428_kcb102_AB-FtsZ_7xR4_50pM_fov1_DP_1_drift_filter_SPOR-picked.hdf5	5.42
230513_Gemini_fov2_Zring_FtsZ_DP_1_drift_picked.hdf5	4.62
230317_fov3_2plex_kcb1113_200pM-r2_DP_FtsZ_1_DRIFT_aligned_picked.hdf5	5.82
042320_fov2_exch_ftsz_dp_1_drift_FtsZ_spor_picked.hdf5	5.27
230513_apollo_Zrings_fov8_r2-300pM_FtsZ_DP_1_drift_picked.hdf5	5.71
230309_kcb1113_veg_2plex_100pM-R2_fov2_FtsZ_DP_1_drift_picked.hdf5	5.64
230401_fov1_kcb1113_150pMr2_FtsZ_DP_1_drift_aligned_picked.hdf5	6.69
230513_Gemini_fov7_Zring_300pM_FtsZ_DP_1_MMStack_Pos0.ome_locs_picked.hdf5	5.59
230310_kcb1113_2plex_spor_fov5_200pM-r2_FtsZ_DP_1_drift_picked.hdf5	8.26
221015_2plex_rab7xR4_gfp5xR1_kcb1113-fov3-FtsZ_1_drift_picked.hdf5	5.17
200619_exchange2_fov1_ftsZ_dp_1_aligned_picked.hdf5	4.85
230513_Gemini_fov7_Zring_300pM_FtsZ_DP_1_MMStack_Pos0.ome_locs_picked.hdf5	5.25
221006_spor_KCB324_rab5xR2_GFP5xR1_fov2_FtsZ_1_aligned_picked.hdf5	5.77
042620_spor_exh_fov2_60min_ftsz_dp_1_drift_early_sporsept.hdf5	5.64
200717_exch_kcb300_fov2_dp_ftsz_1_aligned_picked.hdf5	3.83
230401_fov5_kcb1113_115pMr2_FtsZ_DP_1_MMStack_Pos0.ome_locs_picked.hdf5	7.00
200524_test_2pt0_fov0_dp_ftsz_1_aligned_picked.hdf5	6.23
230317_fov1_2plex_kcb1113_200pM-r2_DP_FtsZ_1_drift_aligned_picked.hdf5	6.19
230513_Gemini_fov8_Zring_300pM_FtsZ_DP_1_drift_picked.hdf5	4.77
230513_apollo_Zrings_fov8_r2-300pM_FtsZ_DP_1_drift_picked.hdf5	5.92
230513_artemis_Zring_fov6_200pM-r2_ftsz_DP_1_drift_picked.hdf5	4.57
230429_fov1_kcb1113_200pM-r2_FtsZ_DP_1_drift_aligned_picked.hdf5	3.65
230513_apollo_Zrings_fov2_r2-200pM_FtsZ_DP_1_drift_picked.hdf5	6.02
230513_Gemini_fov2_Zring_FtsZ_DP_1_drift_picked.hdf5	4.86
230429_fov2_kcb300_200pM-r2_20mW_FtsZ-DP_1_drift_aligned_picked.hdf5	5.14

230513_artemis_Zring_fov10_300pM-r2_ftsz_DP_1_drift_picked.hdf5	4.69
230310_kcb1113_2plex_spor_fov1_100pM-r2_FtsZ_DP_1_drift_aligned_picked.hdf5	5.39
191218_ftsZ_ntsecondary_fov3_dp_r3_antiftsz_100pM_100mWset561_drift - Copy_picked.hdf5	5.61
230513_Gemini_fov8_Zring_300pM_FtsZ_DP_1_drift_picked.hdf5	5.04
230513_Gemini_fov4_Zring_200pM_FtsZ_DP_1_MMStack_Pos0.ome_locs_picked.hdf5	5.50
230513_artemis_Zring_fov9_300pM-r2_ftsz_DP_1_drift_picked.hdf5	4.68
230513_apollo_Zrings_fov6_r2-300pM_FtsZ_DP_1_drift_picked.hdf5	5.38
200716_exch_new_test_fov1_dp_ftsz_1_aligned_picked.hdf5	4.39
230513_apollo_Zrings_fov4_r2-200pM_FtsZ_DP_1_drift_picked.hdf5	6.48
230314_kcb300_spor_RBnb5xr2_200pm-r2_fov3_FtsZ_DP_1_drift_picked.hdf5	4.56
230513_apollo_Zrings_fov3_r2-200pM_FtsZ_DP_1_drift_picked.hdf5	6.15
230429_fov1_kcb300true_200pM-r2_20mW_FtsZ-DP_1_Mdrift_aligned_picked.hdf5	5.25
230513_Gemini_fov5_Zring_300pM_FtsZ_DP_1_drift_picked.hdf5	4.90
230513_artemis_Zring_fov1_125pM-r2_ftsz_DP_2_drift_picked.hdf5	4.77
220805_kcb102_fov1_3plex_nbGFP5xR1_FtsZ-2_1_drift_aligned_picked.hdf5	6.72
200716_exch_new_fov23_dp_ftsz_2_drift_aligned_picked.hdf5	6.52
230429_fov2_kcb300_200pM-r2_20mW_FtsZ-DP_1_drift_aligned_picked.hdf5	5.34
042320_fov1_exch_ftsz_DP_1_1_drift_aligned_picked.hdf5	4.48
230310_kcb1113_2plex_spor_fov3_200pM-r2_FtsZ_DP_1_drift_aligned_picked.hdf5	6.24
200717_exch_kcb300_fov1_dp_ftsz_1_drift_aligned_picked.hdf5	4.27
230513_apollo_Zrings_fov3_r2-200pM_FtsZ_DP_1_drift_picked.hdf5	6.16
230317_fov1_2plex_kcb1113_200pM-r2_DP_FtsZ_1_drift_aligned_picked.hdf5	6.63
221015_2plex_rab7xR4_gfp5xR1_kcb1113-fov1-FtsZ_1_drift_aligned_picked.hdf5	5.53
230429_fov4_kcb1113_400pM-r2_ftsz_dp_2_drift_aligned_picked.hdf5	5.27
230401_fov3_kcb1113_112pMr2_FtsZ_DP_1_drift_aligned_picked.hdf5	6.86
230513_apollo_Zrings_fov2_r2-200pM_FtsZ_DP_1_drift_picked.hdf5	5.59
230401_fov5_kcb1113_115pMr2_FtsZ_DP_1_MMStack_Pos0.ome_locs_picked.hdf5	6.40
230513_apollo_Zrings_fov6_r2-300pM_FtsZ_DP_1_drift_picked.hdf5	5.83
230314_kcb300_spor_RBnb5xr2_200pm-r2_fov3_FtsZ_DP_1_drift_picked.hdf5	4.81
042620_spor_exh_fov5_60min_ftsz_dp_1_drift_FtsZ_prox_picked.hdf5	5.05
230314_kcb300_spor_RBnb5xr2_200pm-r2_fov1_FtsZ_DP_1_drift_picked.hdf5	5.33
200619_exchange2_fov4_ftsz_dp_1_drift_aligned_picked.hdf5	3.62
230513_apollo_Zrings_fov10_r2-300pM_FtsZ_DP_1_drift_picked.hdf5	6.66
230401_fov2_kcb1113_150pMr2_FtsZ_DP_1_drift_picked.hdf5	6.70
200522_test_fov1000_ftsz_1_drift_aligned_picked.hdf5	11.53
230513_apollo_Zrings_fov9_r2-300pM_FtsZ_DP_1_drift_picked.hdf5	6.51
230429_fov2_kcb1113_200pM-r2_FtsZ_DP_1_drift_aligned_picked.hdf5	10.73
230513_apollo_Zrings_fov7_r2-300pM_FtsZ_DP_1_drift_picked.hdf5	5.68

221006_spor_KCB324_rab5xR2_GFP5xR1_fov4_FtsZ_1_drift_picked.hdf5	6.27
230429_fov1_kcb300true_200pM-r2_20mW_FtsZ-DP_1_Mdrift_aligned_picked.hdf5	5.13
200716_exch_old_fov2_dp_ftsz_1_drift_aligned_picked.hdf5	5.26
042320_fov3_exch_ftsz_dp_1_DRIFT_FtsZ_spor_picked.hdf5	4.94
200716_exch_new_fov21_dp_ftsz_1_aligned_picked.hdf5	4.75
230513_apollo_Zrings_fov5_r2-300pM_FtsZ_DP_1_drift_picked.hdf5	6.20
230513_apollo_Zrings_fov1_r2-125pM_FtsZ_DP_1_drift_picked.hdf5	6.53
230401_fov1_kcb1113_150pMr2_FtsZ_DP_1_drift_aligned_picked.hdf5	6.32
230513_Gemini_fov5_Zring_300pM_FtsZ_DP_1_drift_picked.hdf5	5.30
042620_spor_exh_fov3_60min_ftsz_dp_1_drift_ftsz_spor_picked.hdf5	4.77
221006_spor_KCB324_rab5xR2_GFP5xR1_fov4_FtsZ_1_drift_picked.hdf5	6.34
230513_artemis_Zring_fov6_200pM-r2_ftsz_DP_1_drift_picked.hdf5	5.20
042620_spor_exh_fov8_60min_ftsz_dp_1_drift_FtsZ_spor_picked.hdf5	4.63
042620_spor_exh_fov4_60min_ftsz_dp_1_drift_FtsZ_prox_picked.hdf5	5.29
230310_kcb1113_2plex_spor_fov3_200pM-r2_FtsZ_DP_1_drift_aligned_picked.hdf5	4.87
042620_spor_exh_fov6_60min_ftsz_dp_1_drift_FtsZ_prox_picked.hdf5	4.95
230314_kcb300_spor_RBnb5xr2_200pm-r2_fov1_FtsZ_DP_1_drift_picked.hdf5	5.40
200618_exchange1_fov2_redo_ftsz_dp_1_drift_aligned_picked.hdf5	3.28
230429_fov2_kcb1113_200pM-r2_FtsZ_DP_1_drift_aligned_picked.hdf5	4.40
200717_exch_kcb300_fov3_dp_ftsz_1_align_picked.hdf5	4.16
230310_kcb1113_2plex_spor_fov2_160pM-r2_FtsZ_DP_1_aligned_picked.hdf5	5.59
230429_fov4_kcb1113_400pM-r2_ftsz_dp_2_drift_aligned_picked.hdf5	5.39
200717_exch_kcb300_fov5_dp_ftsz_1_DRIFT_aligned_picked.hdf5	4.07
230513_artemis_Zring_fov1_125pM-r2_ftsz_DP_2_drift_picked.hdf5	5.46
200522_fov1002_bf_dp_ftsz_1_drift_aligned_picked.hdf5	6.27
230513_artemis_Zring_fov2_125pM-r2_ftsz_DP_1_drift_picked.hdf5	5.33
221015_2plex_rab7xR4_gfp5xR1_kcb1113-fov1-FtsZ_1_drift_aligned_picked.hdf5	4.89
230513_artemis_Zring_fov10_300pM-r2_ftsz_DP_1_drift_picked.hdf5	4.59
230513_Gemini_fov10_Zring_300pM_FtsZ_DP_1_drift_aligned_picked.hdf5	4.93
042320_fov3_exch_ftsz_dp_1_DRIFT_picked.hdf5	5.53
200619_exchange2_fov2_ftsz_dp_1_drift_aligned_picked.hdf5	4.34
230310_kcb1113_2plex_spor_fov1_100pM-r2_FtsZ_DP_1_drift_aligned_picked.hdf5	5.72
200618_exchange1_fov1_ftsz_DP_1_drift_aligned_picked.hdf5	4.28
230513_artemis_Zring_fov5_200pM-r2_ftsz_DP_1_drift_picked.hdf5	4.75
230314_kcb300_spor_RBnb5xr2_200pm-r2_fov6_FtsZ_DP_1_drift_picked.hdf5	4.85
230314_kcb300_spor_RBnb5xr2_200pm-r2_fov4_FtsZ_DP_1_drift_picked.hdf5	4.66
230429_fov3_kcb300_115pM-r2_20mW_ftsz_DP_2_drift_aligned3_picked.hdf5	7.15
200524_2pt0_fov2_dp_ftsz_1_drift_aligned_picked.hdf5	9.65
220913_kcb306_spor_alfa2xr3_rab5xR2_fov3_200pM-R2_FtsZ_2_drift_picked.hdf5	4.79

230513_Gemini_fov1_Zring_FtsZ_DP_1_drift_picked.hdf5	4.80
221006_spor_KCB324_rab5xR2_GFP5xR1_fov1_FtsZ_DP_1_aligned_PICKED.hdf5	9.12
042320_fov1_exch_ftsz_DP_1_1_drift_FtsZ_spor_picked.hdf5	4.36
230513_apollo_Zrings_fov4_r2-200pM_FtsZ_DP_1_drift_picked.hdf5	6.31
230513_artemis_Zring_fov9_300pM-r2_ftsz_DP_1_drift_picked.hdf5	4.38
230516_artemis_kcb102_fov3_1nM-r3_ZapA_DP_1_drift_aligned_picked.hdf5	10.33
230516_artemis_kcb102_fov1_1nM-R3_ZapA_DP_1_drift_aligned_picked.hdf5	5.85
230510_fov6_kcb328_2per_20mW_1nM-r3_ZapA_DP_1_drift_filter_picked.hdf5	5.61
230510_fov2_kcb328_2per_20mW_1nM-r3_ZapA_DP_2_drift_picked.hdf5	7.43
230516_artemis_kcb102_fov3_1nM-r3_ZapA_DP_1_drift_aligned_picked.hdf5	7.95
230510_fov2_kcb328_2per_20mW_1nM-r3_ZapA_DP_2_drift_picked.hdf5	7.70
230510_fov4_kcb328_2per_20mW_1nM-r3_ZapA_DP_1_drift_aligned_filter_picked.hdf5	5.58
230512_fov2_kcb102_1nM-R3_ZapA_DP_1_drift_aligned_filter_picked.hdf5	5.99
230516_artemis_kcb102_fov1_1nM-R3_ZapA_DP_1_drift_aligned_picked.hdf5	6.12
230512_fov2_kcb102_1nM-R3_ZapA_DP_1_drift_aligned_filter_picked.hdf5	6.63
230512_fov1_kcb102_1nM-R3_ZapA_DP_1_drift_aligned_filter_picked.hdf5	8.26
220805_kcb102_fov1_3plex_ALFA2xR3_ZapA_1_drift_aligned_picked.hdf5	6.28
230510_fov6_kcb328_2per_20mW_1nM-r3_ZapA_DP_1_drift_filter_picked.hdf5	4.82
230516_artemis_kcb102_fov4_1nM-r3_ZapA_DP_1_drift_aligned_filter_picked.hdf5	8.39
230510_fov4_kcb328_2per_20mW_1nM-r3_ZapA_DP_1_drift_aligned_filter_picked.hdf5	6.05
230516_artemis_kcb102_fov4_1nM-r3_ZapA_DP_1_drift_aligned_filter_picked.hdf5	7.52
220721_kcb102_spor_ZapA_2xR3_fov2-DP_1_drift_picked.hdf5	7.16
220805_kcb102_fov1_3plex_ALFA2xR3_ZapA_1_drift_aligned_picked.hdf5	6.12
230512_fov1_kcb102_1nM-R3_ZapA_DP_1_drift_aligned_filter_picked.hdf5	8.45
042620_spor_exh_fov3_60min_div_dp_1_drift_aligned_picked.hdf5	6.89
042620_spor_exh_fov3_60min_div_dp_1_drift_aligned_picked.hdf5	5.42
200619_exchange2_fov2_ftsz_dp_1_drift_aligned_picked.hdf5	4.03
042320_fov1_exch_DIV_DP_1_1_drift_aligned_picked_DivIVA_avg3.hdf5	4.41
200717_exch_kcb300_fov2_dp_ftsz_1_aligned_picked_avg3.hdf5	6.85
042620_spor_exh_fov8_60min_ftsz_dp_1_aligned_picked_ftsz.hdf5	4.27
042620_spor_exh_fov8_60min_ftsz_dp_1_aligned_picked_avg3.hdf5	4.27
200618_exchange1_fov1_DivIVA_DP_1_drift_aligned_picked.hdf5	6.20
200717_exch_kcb300_fov2_dp_ftsz_1_aligned_picked_avg3.hdf5	9.70
200717_exch_kcb300_fov5_dp_DivIVA_1_DRIFT_aligned_picked_Div.hdf5	5.69
200717_exch_kcb300_fov2_dp_ftsz_1_aligned_picked.hdf5	9.71
200717_exch_kcb300_fov1_dp_DivIVA_1_drift_aligned.hdf5	4.01
200618_exchange1_fov1_DivIVA_DP_1_drift_aligned_picked_avg3.hdf5	5.19

042620_spor_exh_fov3_60min_div_dp_1_drift_aligned_picked_avg3.hdf5	5.42
200717_exch_kcb300_fov4_dp_diviva_1_drift_align_picked.hdf5	9.09
042320_fov1_exch_DIV_DP_1_1_drift_aligned_picked_DivIVA.hdf5	4.41
200717_exch_kcb300_fov2_dp_ftsz_1_aligned_picked.hdf5	6.85
200618_exchange1_fov1_ftsz_DP_1_drift_aligned_picked_avg3.hdf5	4.67
042620_spor_exh_fov8_60min_diviva_dp_1_aligned_picked.hdf5	7.96
200619_exchange2_fov2_ftsz_dp_1_drift_aligned_picked_avg3.hdf5	4.03
200717_exch_kcb300_fov5_dp_DivIVA_1_DRIFT_aligned_picked_Ftsz.hdf5	11.00
042620_spor_exh_fov8_60min_diviva_dp_1_aligned_picked_avg3.hdf5	7.96
200717_exch_kcb300_fov1_dp_DivIVA_1_drift_aligned_avg3.hdf5	5.80
042620_spor_exh_fov8_60min_diviva_dp_1_aligned_picked_Div.hdf5	7.81
200619_exchange2_fov2_diviva_dp_1_drift_aligned_picked_avg3.hdf5	3.88
042320_fov1_exch_ftsz_DP_1_1_drift_aligned_picked_FtsZ_avg3.hdf5	3.00
200717_exch_kcb300_fov4_dp_diviva_1_drift_align_picked_avg3.hdf5	9.09
042620_spor_exh_fov3_60min_div_dp_1_drift_aligned_picked_avg3.hdf5	6.89
042320_fov1_exch_ftsz_DP_1_1_drift_aligned_picked_FtsZ.hdf5	3.00
200717_exch_kcb300_fov4_dp_ftsz_1_drift_align_picked.hdf5	5.11
200717_exch_kcb300_fov1_dp_DivIVA_1_drift_aligned.hdf5	5.80
200618_exchange1_fov1_ftsz_DP_1_drift_aligned_picked.hdf5	4.67
200619_exchange2_fov2_diviva_dp_1_drift_aligned_picked.hdf5	3.88
042620_spor_exh_fov8_60min_ftsz_dp_1_aligned_picked.hdf5	4.27
200717_exch_kcb300_fov1_dp_DivIVA_1_drift_aligned_avg3.hdf5	4.01
200717_exch_kcb300_fov4_dp_ftsz_1_drift_align_picked_avg3.hdf5	5.12
200203_CONDTN2_fov1_diviva_1_drift_Picklocs.hdf5	9.45
042620_spor_exh_fov3_60min_div_dp_1_drift_DivIVA_dualprox.hdf5	5.74
042620_spor_exh_fov1_15min_diviva__1_drift_DivIVA_dualprox_picked.hdf5	6.48
042320_fov2_exch_div_dp_1_DRIFT_diviva_dualprox_picked.hdf5	5.62
042320_fov1_exch_DIV_DP_1_1_DRIFT_Div_dualprox_picked.hdf5	4.56
042620_spor_exh_fov8_60min_diviva_dp_1_drift_DivIVA_dualprox_picked.hdf5	5.74
200220_Round5_kcb300_fov2_diviva_1_DRFIT_dualDivprox_picked.hdf5	6.45
200130_kcb300_fov1_DIVIVA_1_drift_SingleCell_DivIVAprox_picked.hdf5	7.00
042620_spor_exh_fov2_60min_diviva_dp_1_rift_DivIVA_dualprox_picked.hdf5	6.35
200203_CONDTN1_fov2_diviva_1_MMStack_Pos0.ome_locs_dualDivprox_picked.hdf5	13.40
042620_spor_exh_fov4_60min_div_dp_1_drift_DivIVA_Dualprox_picked.hdf5	7.51
042620_spor_exh_fov6_60min_div_1_drift_DivIVA_dualprox_picked.hdf5	5.14
042320_fov3_exch_div_dp_1_DRIFT_DivIVA_dualprox_picked.hdf5	4.92
042320_fov24_DIVIVAonly_multifov_dp_1_driftr_DivIVA_dualprox_picked.hdf5	6.49
042320_fov24_DIVIVAonly_multifov_dp_1_MMStack_fov20.ome_DivIVA_dualprox_picked.hdf5	5.53

200220_Round6_kcb300_fov1_R1diviva_1_DRIFT_singlecell_1DivProx_LocsPicked.hdf5	5.39
042220_kcb300_spor_dp__1_MMStack_fov100.ome_DRIFT_DivIVA_dualprox_picked.hdf5	5.98
042220_kcb300_spor_dp__1_MMStack_fov102.ome_DRIFT_DivIVA_dualproxpicked.hdf5	5.69
042620_spor_exh_fov5_60min_diviva_dp_1_drift_DivIVA_dualprox_picked.hdf5	6.84
200717_exch_kcb300_fov1_dp_DivIVA_1_drift_aligned_picked.hdf5	6.22
200716_exch_new_test_fov1_dp_DivIVA_1_aligned_picked.hdf5	6.83
200717_exch_kcb300_fov4_dp_DivIVA_1_drift_align_picked.hdf5	7.33
200619_exchange2_fov1_DivIVA_dp_1_aligned_picked.hdf5	6.06
042620_spor_exh_fov2_60min_DivIVA_aligned_picked.hdf5	6.31
042620_spor_exh_fov6_60min_DivIVA_dp_1_drift_aligned_picked.hdf5	5.13
200522_test_fov1000_DivIVA_1_drift_aligned_picked.hdf5	6.96
042320_fov3_exch_DivIVA_dp_1_DRIFT_align_picked.hdf5	4.44
042620_spor_exh_fov4_60min_DivIVA_dp_picked.hdf5	7.55
200717_exch_kcb300_fov2_dp_DivIVA_1_aligned_picked.hdf5	5.97
200618_exchange1_fov2_redo_DivIVA_dp_1_drift_aligned_picked.hdf5	4.72
200618_exchange1_fov7_DivIVA_dp_1_drift_aligned_picked.hdf5	7.98
200717_exch_kcb300_fov3_dp_DivIVA_1_align_picked.hdf5	5.54
200619_exchange2_fov2_DivIVA_dp_1_drift_aligned_picked.hdf5	4.63
200618_exchange1_fov1_DivIVA_DP_1_drift_aligned_picked.hdf5	5.68
200203_CONDTN2_fov1_DivIVA_1_drift_aligned_picked.hdf5	9.37
042620_spor_exh_fov5_60min_DivIVA_dp_1_picked.hdf5	7.12
200716_exch_new_fov21_dp_DivIVA_1_aligned_picked.hdf5	6.92
042320_fov2_exch_DivIVA_dp_1_drift_aligned_picked.hdf5	6.34
042620_spor_exh_fov8_60min_DivIVA_dp_1_aligned_picked.hdf5	6.06
200716_exch_new_fov23_dp_DivIVA_2_drift_aligned_picked.hdf5	9.04
200716_exch_old_test_fov1_dp_1_DivIVA_aligned_picked.hdf5	5.10
200716_exch_new_fov20_dp_DivIVA_1_aligned_picked.hdf5	6.03
200524_test_2pt0_fov0_dp_DivIVA_1_aligned_picked.hdf5	7.08
200716_exch_old_fov2_dp_DivIVA_1_drift_aligned_picked.hdf5	5.32
200524_2pt0_fov2_dp_diviva_1_drift_aligned_picked.hdf5	4.62
200524_test_0p75_fov0_dp_DivIVA_1_aligned_picked.hdf5	6.56
200717_exch_kcb300_fov5_dp_DivIVA_1_DRIFT_aligned_picked.hdf5	5.80
200619_exchange2_fov4_DivIVA_dp_1_drift_aligned_picked.hdf5	3.76
042620_spor_exh_fov3_60min_DivIVA_picked.hdf5	5.81
200522_fov1002_bf_dp_DivIVA_1_drift_aligned_picked.hdf5	8.15
200717_exch_kcb300_fov2_dp_ftsz_1_aligned_picked.hdf5	3.83
200717_exch_kcb300_fov3_dp_ftsz_1_align_picked.hdf5	4.16

042620_spor_exh_fov5_60min_ftsz_dp_1_picked.hdf5	5.57
042320_fov2_exch_ftsz_dp_1_drift_aligned_picked.hdf5	5.37
042320_fov3_exch_ftsz_dp_1_DRIFT_align_picked.hdf5	4.42
042620_spor_exh_fov2_60min_ftsz_aligned_picked.hdf5	5.14
200524_test_0p75_fov0_dp_ftsz_1_aligned_picked.hdf5	4.98
200716_exch_new_test_fov1_dp_ftsz_1_aligned_picked.hdf5	4.39
042620_spor_exh_fov8_60min_ftsz_dp_1_aligned_picked.hdf5	4.86
200716_exch_new_fov20_dp_ftsz_1_aligned_picked.hdf5	5.19
042620_spor_exh_fov3_60min_ftsz_picked.hdf5	4.48
200524_test_2pt0_fov0_dp_ftsz_1_aligned_picked.hdf5	6.23
200203_CONDTN2_fov1_ftsz_1_drift_aligned_picked.hdf5	6.21
200619_exchange2_fov2_ftsz_dp_1_drift_aligned_picked.hdf5	4.34
200618_exchange1_fov8_ftsz_dp_1_aligned_picked.hdf5	4.37
200618_exchange1_fov2_redo_ftsz_dp_1_drift_aligned_picked.hdf5	3.28
200716_exch_new_fov21_dp_ftsz_1_aligned_picked.hdf5	4.75
200618_exchange1_fov1_ftsz_DP_1_drift_aligned_picked.hdf5	4.28
200716_exch_old_test_fov1_dp_1_ftsz_aligned_picked.hdf5	5.11
200716_exch_new_fov23_dp_ftsz_2_drift_aligned_picked.hdf5	6.52
200619_exchange2_fov4_ftsz_dp_1_drift_aligned_picked.hdf5	3.62
200717_exch_kcb300_fov4_dp_ftsz_1_drift_align_picked.hdf5	4.34
042620_spor_exh_fov4_60min_ftsz_dp_picked.hdf5	5.15
200717_exch_kcb300_fov1_dp_ftsz_1_drift_aligned_picked.hdf5	4.27
200717_exch_kcb300_fov5_dp_ftsz_1_DRIFT_aligned_picked.hdf5	4.07
200524_2pt0_fov2_dp_ftsz_1_drift_aligned_picked.hdf5	9.65
200619_exchange2_fov1_ftsZ_dp_1_aligned_picked.hdf5	4.85
200716_exch_old_fov2_dp_ftsz_1_drift_aligned_picked.hdf5	5.26
200522_fov1002_bf_dp_ftsz_1_drift_aligned_picked.hdf5	6.27
042620_spor_exh_fov6_60min_ftsz_dp_1_drift_aligned_picked.hdf5	4.21
200618_exchange1_fov7_ftsz_dp_1_drift_aligned_picked.hdf5	4.90
200522_test_fov1000_ftsz_1_drift_aligned_picked.hdf5	11.53
042620_spor_exh_fov3_60min_DivIVA_dp_1_drift_aligned_picklocs.hdf5	6.92
200716_exch_old_fov2_dp_DivIVA_1_drift_aligned_picklocs.hdf5	5.61
200716_exch_new_fov23_dp_DivIVA_1_aligned_picklocs.hdf5	9.19
042320_fov3_exch_DivIVA_dp_1_DRIFT_align_picked.hdf5	4.97
200717_exch_kcb300_fov2_dp_DivIVA_1_drift_aligned_picklocs.hdf5	6.34
200203_CONDTN2_fov1_diviva_1_drift_veg_picklocs.hdf5	11.02
200716_exch_old_test_fov1_dp_1_DivIVA_drift_aligned_picklocs.hdf5	6.11
200618_exchange1_fov2_redo_DivIVA_dp_1_aligned_pickregs.hdf5	5.73
200213_fov1_200pmR1_DivIVA_1_drift_veg_picklocs.hdf5	9.98

200717_exch_kcb300_fov4_dp_DivIVA_1_align_picklocs.hdf5	11.72
042320_fov2_exch_DivIVA_dp_1_DRIFT_aligned_picklocs.hdf5	6.29
042620_spor_exh_fov6_60min_DivIVA_dp_1_drift_aligned_picklocs.hdf5	5.59
200130_kcb300_fov1_DIVIVA_1_drift_veg_picklocs.hdf5	8.08
200717_exch_kcb300_fov5_dp_DivIVA_1_DRIFT_aligned_pickregs.hdf5	5.40
042320_fov1_exch_DivIVA_DP_1_1_DRIFT_aligned_picked.hdf5	4.84
042620_spor_exh_fov4_60min_DivIVA_dp_1_aligned_picklocs.hdf5	8.38
200716_exch_new_test_fov1_DivIVA_2_drift_aligned_picklocs.hdf5	7.56
200618_exchange1_fov1_DivIVA_DP_1_drift_aligned_picklocs.hdf5	5.24
200717_exch_kcb300_fov3_dp_DivIVA_1_drift_align_pickregs.hdf5	5.21
200716_exch_new_fov20_dp_DivIVA_1_drift_aligned_picklocs.hdf5	8.16
200717_exch_kcb300_fov6_dp_DivIVA_1_aligned_picklocs.hdf5	7.93
230530_kcb102_Gemini_fov1_2plex_r4-150pM_FtsZ_DP_2_drift_aligned_picked.hdf5	4.23
230516_artemis_kcb102_fov3_180pM-r5_FtsZ_DP_1_drift_aligned_picked.hdf5	7.23
230530_kcb102_Gemini_fov1_2plex_r3-800pM_ZapA_DP_1_drift_aligned_picked.hdf5	4.75
230516_artemis_kcb102_fov1_1nM-R3_ZapA_DP_1_drift_aligned_picked.hdf5	5.63
230516_artemis_kcb102_fov3_1nM-r3_ZapA_DP_1_drift_aligned_picked.hdf5	9.90
230517_apollo_kcb102_fov4_r5-180pM_FtsZ_DP_1_drift_aligned_picked.hdf5	6.12
230516_artemis_kcb102_fov1_1nM-R3_ZapA_DP_1_drift_aligned_picked.hdf5	5.90
230512_fov1_kcb102_1nM-R3_ZapA_DP_1_drift_aligned_ZapA_picked.hdf5	7.68
230516_artemis_kcb102_fov4_1nM-r3_ZapA_DP_1_drift_aligned_picked.hdf5	7.90
230512_fov2_kcb102_1nM-R3_ZapA_DP_1_drift_aligned_filter_picked.hdf5	6.66
230512_fov2_kcb102_125pM-R2_FtsZ_DP_1_drift_aligned_picked.hdf5	6.12
230512_fov2_kcb102_1nM-R3_ZapA_DP_1_drift_aligned_filter_picked.hdf5	6.33
230516_artemis_kcb102_fov1_180pM-R5_FtsZ_DP_1_drift_aligned_picked.hdf5	5.83
230512_fov1_kcb102_1nM-R3_ZapA_DP_1_drift_aligned_ZapA_picked.hdf5	8.11
230516_artemis_kcb102_fov3_180pM-r5_FtsZ_DP_1_drift_aligned_picked.hdf5	7.92
230512_fov1_kcb102_125pM-R4_FtsZ_DP_1_drift_aligned_FtsZ_picked.hdf5	6.61
230516_artemis_kcb102_fov3_1nM-r3_ZapA_DP_1_drift_aligned_picked.hdf5	8.05
230516_artemis_kcb102_fov4_1nM-r3_ZapA_DP_1_drift_aligned_filter_picked.hdf5	7.52
230516_artemis_kcb102_fov4_180pM-r5_FtsZ_DP_1_drift_aligned_picked.hdf5	6.90
230517_apollo_kcb102_fov4_r3-1nM_ZapA_DP_1_drift_aligned_filter_picked.hdf5	6.94
230517_apollo_kcb102_fov4_r3-1nM_ZapA_DP_1_drift_aligned_filter_picked.hdf5	6.85
230516_artemis_kcb102_fov1_180pM-R5_FtsZ_DP_1_drift_aligned_picked.hdf5	5.77
230517_apollo_kcb102_fov4_r5-180pM_FtsZ_DP_1_drift_aligned_picked.hdf5	6.71
230512_fov1_kcb102_125pM-R4_FtsZ_DP_1_drift_aligned_FtsZ_picked.hdf5	7.21
230512_fov2_kcb102_125pM-R2_FtsZ_DP_1_drift_aligned_picked.hdf5	6.25
230621_apollo_kcb328_fov1_2plex_r4-170pM_DP_FtsZ_1_drift_aligned_picked.hdf5	5.73
230621_apollo_kcb328_fov1_2plex_r3-1nM_DP_ZapA_1_drift_aligned_picked.hdf5	7.28

230530_kcb102_Gemini_fov1_2plex_r4-150pM_FtsZ_DP_2_drift_aligned_picked.hdf5	4.23
230530_kcb102_Gemini_fov1_2plex_r3-800pM_ZapA_DP_1_drift_aligned_picked.hdf5	4.75
230530_kcb102_Gemini_fov3_2plex_r4-170pM_FtsZ_DP_1drift_aligned_picked.hdf5	4.91
230530_kcb102_Gemini_fov3_2plex_r3-800pM_ZapA_DP_1_drift_aligned_picked.hdf5	5.79
230621_kcb328_veg_fov1_r4-170pM_DP_FtsZ_1_drift_aligned_picked.hdf5	4.04
230621_kcb328_veg_fov1_2_r3-1nM_DP_ZapA_1_drift_aligned_picked.hdf5	5.60
230530_kcb102_Gemini_fov2_2plex_r3-800pM_ZapA_DP_1_drift_aligned_picked.hdf5	6.44
230530_kcb102_Gemini_fov2_2plex_r4-170pM_FtsZ_DP_1_drift_aligned_picked.hdf5	4.85
230401_fov2_kcb1113_50pMr2_SepF_DP_1_drift_aligned_picked.hdf5	6.21
230518_artemis_kcb1113_fov3_150pM-r2_SepF_DP_1_drift_aligned_picked.hdf5	5.05
230518_artemis_kcb1113_fov2_180pM-r5_FtsZ_DP_1_aligned_picked.hdf5	5.52
230518_artemis_kcb1113_fov3_150pM-r2_SepF_DP_1_drift_aligned_picked.hdf5	4.63
230518_artemis_kcb1113_fov3_180pM-r5_FsZ_DP_1_drift_aligned_picked.hdf5	4.43
230518_artemis_kcb1113_fov4_180pM-r5_FsZ_DP_1_drift_aligned_picked.hdf5	4.40
230310_kcb1113_2plex_spor_fov3_500pM-r3_SepF_DP_1_drift_aligned_picked.hdf5	5.05
230401_fov2_kcb1113_150pMr2_FtsZ_DP_1_drift_aligned_picked.hdf5	6.46
230401_fov2_kcb1113_50pMr2_SepF_DP_1_drift_aligned_picked.hdf5	6.86
230518_artemis_kcb1113_fov1_150pM-r2_DP-2_1_drift_aligned_picked.hdf5	4.78
230518_artemis_kcb1113_fov4_140pM-r2_SepF_DP_1_drift_aligned_picked.hdf5	5.08
230518_artemis_kcb1113_fov1_180pM-r5_DP-2_1_drift_aligned_picked.hdf5	4.80
230401_fov1_kcb1113_500pMr4_SepF_DP_1_drift_filter-2_aligned_picked.hdf5	6.55
230401_fov2_kcb1113_150pMr2_FtsZ_DP_1_drift_aligned_picked.hdf5	7.28
230401_fov3_kcb1113_500pMr2_SepF_DP_1_drift_aligned_filter_picked.hdf5	6.23
230518_artemis_kcb1113_fov2_150pM-r2_SepF_DP_1_drift_aligned_picked.hdf5	4.80
230518_artemis_kcb1113_fov1_150pM-r2_DP-2_1_drift_aligned_picked.hdf5	5.36
230401_fov3_kcb1113_112pMr2_FtsZ_DP_1_drift_aligned_picked.hdf5	6.76
230317_fov1_2plex_kcb1113_375pM-r3_DP_1_drift_aligned_picked.hdf5	5.19
230518_artemis_kcb1113_fov4_140pM-r2_SepF_DP_1_drift_aligned_picked.hdf5	4.32
230310_kcb1113_2plex_spor_fov3_500pM-r3_SepF_DP_1_drift_aligned_picked.hdf5	4.70
230518_artemis_kcb1113_fov1_180pM-r5_DP-2_1_drift_aligned_picked.hdf5	4.77
230401_fov1_kcb1113_150pMr2_FtsZ_DP_1_drift_aligned_picked.hdf5	7.41
230317_fov1_2plex_kcb1113_200pM-r2_DP_1_drift_aligned_picked.hdf5	6.16
230401_fov3_kcb1113_112pMr2_FtsZ_DP_1_drift_aligned_picked.hdf5	6.35
230518_artemis_kcb1113_fov2_180pM-r5_FtsZ_DP_1_aligned_picked.hdf5	5.83
230310_kcb1113_2plex_spor_fov3_200pM-r2_FtsZ_DP_1_drift_aligned_picked.hdf5	4.72
230310_kcb1113_2plex_spor_fov3_200pM-r2_FtsZ_DP_1_drift_aligned_picked.hdf5	5.21
230317_fov1_2plex_kcb1113_375pM-r3_DP_1_drift_aligned_picked.hdf5	5.32
230401_fov1_kcb1113_500pMr4_SepF_DP_1_drift_filter-2_aligned_picked.hdf5	7.79
230518_artemis_kcb1113_fov3_180pM-r5_FsZ_DP_1_drift_aligned_picked.hdf5	4.48

230401_fov1_kcb1113_150pMr2_FtsZ_DP_1_drift_aligned_picked.hdf5	6.43
230401_fov3_kcb1113_500pMr2_SepF_DP_1_drift_aligned_filter_picked.hdf5	6.14
230518_artemis_kcb1113_fov2_150pM-r2_SepF_DP_1_drift_aligned_picked.hdf5	5.04
230317_fov1_2plex_kcb1113_200pM-r2_DP_1_drift_aligned_picked.hdf5	6.63
230518_artemis_kcb1113_fov4_180pM-r5_FsZ_DP_1_drift_aligned_picked.hdf5	3.94

REFERENCES

1. E. F. Bi, J. Lutkenhaus, FtsZ ring structure associated with division in Escherichia coli. *Nature* **354**, 161–164 (1991).

2. J. Errington, L. J. Wu, Cell cycle machinery in *Bacillus subtilis*. *Subcell. Biochem.* **84**, 67–101 (2017).

3. P. Gamba, J. W. Veening, N. J. Saunders, L. W. Hamoen, R. A. Daniel, Two-step assembly dynamics of the *Bacillus subtilis* divisome. *J. Bacteriol.* **191**, 4186–4194 (2009).

4. L. G. Monahan, A. Robinson, E. J. Harry, Lateral FtsZ association and the assembly of the cytokinetic Z ring in bacteria. *Mol. Microbiol.* **74**, 1004–1017 (2009).

5. R. Duman, S. Ishikawa, I. Celik, H. Strahl, N. Ogasawara, P. Troc, J. Lowe, L. W. Hamoen, Structural and genetic analyses reveal the protein SepF as a new membrane anchor for the Z ring. *Proc. Natl. Acad. Sci. U.S.A.* **110**, E4601–E4610 (2013).

6. P. Szwedziak, Q. Wang, T. A. Bharat, M. Tsim, J. Lowe, Architecture of the ring formed by the tubulin homologue FtsZ in bacterial cell division. *eLife* **3**, e04601 (2014).

7. T. Mohammadi, G. E. Ploeger, J. Verheul, A. D. Comvalius, A. Martos, C. Alfonso, J. van Marle, G. Rivas, T. den Blaauwen, The GTPase activity of Escherichia coli FtsZ determines the magnitude of the FtsZ polymer bundling by ZapA in vitro. *Biochemistry* **48**, 11056–11066 (2009).

8. S. O. Jensen, L. S. Thompson, E. J. Harry, Cell division in *Bacillus subtilis*: FtsZ and FtsA association is Z-ring independent, and FtsA is required for efficient midcell Z-Ring assembly. *J. Bacteriol.* **187**, 6536–6544 (2005).

9. S. Pichoff, J. Lutkenhaus, Tethering the Z ring to the membrane through a conserved membrane targeting sequence in FtsA. *Mol. Microbiol.* **55**, 1722–1734 (2005).

10. N. Pende, A. Sogues, D. Megrian, A. Sartori-Rupp, P. England, H. Palabikyan, S. K. R. Rittmann, M. Grana, A. M. Wehenkel, P. M. Alzari, S. Gribaldo, SepF is the FtsZ anchor in archaea, with features of an ancestral cell division system. *Nat. Commun.* **12**, 3214 (2021).

11. M. Wenzel, I. N. Celik Gulsoy, Y. Gao, Z. Teng, J. Willemse, M. Middelkamp, M. G. M. van Rosmalen, P. W. B. Larsen, N. N. van der Wel, G. J. L. Wuite, W. H. Roos, L. W. Hamoen, Control of septum thickness by the curvature of SepF polymers. *Proc. Natl. Acad. Sci. U.S.A.* **118**, e2002635118 (2021).

12. K. Khanna, J. Lopez-Garrido, J. Sugie, K. Pogliano, E. Villa, Asymmetric localization of the cell division machinery during *Bacillus subtilis* sporulation. *eLife* **10**, e62204 (2021).

13. P. Eswaramoorthy, P. W. Winter, P. Wawrzusin, A. G. York, H. Shroff, K. S. Ramamurthi, Asymmetric division and differential gene expression during a bacterial developmental program requires DivIVA. *PLOS Genet.* **10**, e1004526 (2014).

14. J. H. Cha, G. C. Stewart, The divIVA minicell locus of *Bacillus subtilis*. *J. Bacteriol.* **179**, 1671–1683 (1997).

15. D. H. Edwards, J. Errington, The *Bacillus subtilis* DivIVA protein targets to the division septum and controls the site specificity of cell division. *Mol. Microbiol.* **24**, 905–915 (1997).

16. I. Barak, P. Prepiak, F. Schmeisser, MinCD proteins control the septation process during sporulation of *Bacillus subtilis*. *J. Bacteriol.* **180**, 5327–5333 (1998).

17. A. Lablaine, M. Serrano, C. Bressuire-Isoard, S. Chamot, I. Bornard, F. Carlin, A. O. Henriques, V. Broussolle, The morphogenetic protein CotE positions exosporium proteins CotY and ExsY during sporulation of Bacillus cereus. *mSphere* **6**, e00007-21 (2021).

18. J. Schnitzbauer, M. T. Strauss, T. Schlichthaerle, F. Schueder, R. Jungmann, Super-resolution microscopy with DNA-PAINT. *Nat. Protoc.* **12**, 1198–1228 (2017).

19. R. Jungmann, M. S. Avendano, M. Dai, J. B. Woehrstein, S. S. Agasti, Z. Feiger, A. Rodal, P. Yin, Quantitative super-resolution imaging with qPAINT. *Nat. Methods* **13**, 439–442 (2016).

20. R. Jungmann, M. S. Avendano, J. B. Woehrstein, M. Dai, W. M. Shih, P. Yin, Multiplexed 3D cellular super-resolution imaging with DNA-PAINT and Exchange-PAINT. *Nat. Methods* **11**, 313–318 (2014).

21. H. Gotzke, M. Kilisch, M. Martinez-Carranza, S. Sograte-Idrissi, A. Rajavel, T. Schlichthaerle, N. Engels, R. Jungmann, P. Stenmark, F. Opazo, S. Frey, The ALFA-tag is a highly versatile tool for nanobody-based bioscience applications. *Nat. Commun.* **10**, 4403 (2019).

22. M. E. Gundogdu, Y. Kawai, N. Pavlendova, N. Ogasawara, J. Errington, D. J. Scheffers, L. W. Hamoen, Large ring polymers align FtsZ polymers for normal septum formation. *EMBO J.* **30**, 617–626 (2011).

23. A. W. Bisson-Filho, Y. P. Hsu, G. R. Squyres, E. Kuru, F. Wu, C. Jukes, Y. Sun, C. Dekker, S. Holden, M. S. VanNieuwenhze, Y. V. Brun, E. C. Garner, Treadmilling by FtsZ filaments drives peptidoglycan synthesis and bacterial cell division. *Science* **355**, 739–743 (2017).

24. X. Yang, Z. Lyu, A. Miguel, R. McQuillen, K. C. Huang, J. Xiao, GTPase activity-coupled treadmilling of the bacterial tubulin FtsZ organizes septal cell wall synthesis. *Science* **355**, 744–747 (2017).

25. R. Pacheco-Gomez, X. Cheng, M. R. Hicks, C. J. Smith, D. I. Roper, S. Addinall, A. Rodger, T. R. Dafforn, Tetramerization of ZapA is required for FtsZ bundling. *Biochem. J.* **449**, 795–802 (2013).

26. P. Eswaramoorthy, M. L. Erb, J. A. Gregory, J. Silverman, K. Pogliano, J. Pogliano, K. S. Ramamurthi, Cellular architecture mediates DivIVA ultrastructure and regulates min activity in *Bacillus subtilis*. *MBio* **2**, e00257-11 (2011).

27. P. Guzman, J. Westpheling, P. Youngman, Characterization of the promoter region of the *Bacillus subtilis* spoIIE operon. *J. Bacteriol.* **170**, 1598–1609 (1988).

28. I. Barak, P. Youngman, SpoIIE mutants of *Bacillus subtilis* comprise two distinct phenotypic classes consistent with a dual functional role for the SpoIIE protein. *J. Bacteriol.* **178**, 4984–4989 (1996).

29. P. Caldas, M. Lopez-Pelegrin, D. J. G. Pearce, N. B. Budanur, J. Brugues, M. Loose, Cooperative ordering of treadmilling filaments in cytoskeletal networks of FtsZ and its crosslinker ZapA. *Nat. Commun.* **10**, 5744 (2019).

30. L. W. Hamoen, J. C. Meile, W. de Jong, P. Noirot, J. Errington, SepF, a novel FtsZ-interacting protein required for a late step in cell division. *Mol. Microbiol.* **59**, 989–999 (2006).

31. K. S. Ramamurthi, R. Losick, Negative membrane curvature as a cue for subcellular localization of a bacterial protein. *Proc. Natl. Acad. Sci. U.S.A.* **106**, 13541–13545 (2009).

32. J. Pogliano, N. Osborne, M. D. Sharp, A. Abanes-De Mello, A. Perez, Y. L. Sun, K. Pogliano, A vital stain for studying membrane dynamics in bacteria: A novel mechanism controlling septation during *Bacillus subtilis* sporulation. *Mol. Microbiol.* **31**, 1149–1159 (1999).

33. S. van Baarle, M. Bramkamp, The MinCDJ system in *Bacillus subtilis* prevents minicell formation by promoting divisome disassembly. *PLOS ONE* **5**, e9850 (2010).

34. E. Cendrowicz, S. P. van Kessel, L. S. van Bezouwen, N. Kumar, E. J. Boekema, D. J. Scheffers, *Bacillus subtilis* SepF binds to the C-terminus of FtsZ. *PLOS One* **7**, e43293 (2012).

35. R. M. Cleverley, J. R. Barrett, A. Basle, N. K. Bui, L. Hewitt, A. Solovyova, Z. Q. Xu, R. A. Daniel, N. E. Dixon, E. J. Harry, A. J. Oakley, W. Vollmer, R. J. Lewis, Structure and function of a spectrin-like regulator of bacterial cytokinesis. *Nat. Commun.* **5**, 5421 (2014).

36. G. A. Wilson, K. F. Bott, Nutritional factors influencing the development of competence in the *Bacillus subtilis* transformation system. *J. Bacteriol.* **95**, 1439–1449 (1968).

37. J. M. Sterlini, J. Mandelstam, Commitment to sporulation in *Bacillus subtilis* and its relationship to development of actinomycin resistance. *Biochem. J.* **113**, 29–37 (1969).

38. D. G. Gibson, L. Young, R. Y. Chuang, J. C. Venter, C. A. Hutchison, 3rd, H. O. Smith, Enzymatic assembly of DNA molecules up to several hundred kilobases. *Nat. Methods* **6**, 343–345 (2009).

39. K. Cramer, A. L. Bolender, I. Stockmar, R. Jungmann, R. Kasper, J. Y. Shin, Visualization of bacterial protein complexes labeled with fluorescent proteins and nanobody binders for STED microscopy. *Int. J. Mol. Sci.* **20**, 3376 (2019).

40. S. Sograte-Idrissi, N. Oleksiievets, S. Isbaner, M. Eggert-Martinez, J. Enderlein, R. Tsukanov, F. Opazo, Nanobody detection of standard fluorescent proteins enables multi-target DNA-PAINT with high resolution and minimal displacement errors. *Cell* **8**, 48 (2019).

41. U. Endesfelder, S. Malkusch, F. Fricke, M. Heilemann, A simple method to estimate the average localization precision of a single-molecule localization microscopy experiment. *Histochem. Cell Biol.* **141**, 629–638 (2014).

42. D. Comaniciu, P. Meer, Mean shift: A robust approach toward feature space analysis. *IEEE Trans. Pattern Anal. Mach. Intell.* **24**, 603–619 (2002).

43. F. Pedregosa, G. Varoquaux, A. Gramfort, V. Michel, B. Thirion, O. Grisel, M. Blondel, P. Prettenhofer, R. Weiss, V. Dubourg, J. Vanderplas, A. Passos, D. Cournapeau, M. Brucher, M. Perrot, E. Duchesnay, Scikit-learn: Machine learning in Python. *J Mach. Learn. Res.* **12**, 2825–2830 (2011).

44. P. Virtanen, R. Gommers, T. E. Oliphant, M. Haberland, T. Reddy, D. Cournapeau, E. Burovski, P. Peterson, W. Weckesser, J. Bright, S. J. van der Walt, M. Brett, J. Wilson, K. J. Millman, N. Mayorov, A. R. J. Nelson, E. Jones, R. Kern, E. Larson, C. J. Carey, I. Polat, Y. Feng, E. W. Moore, J. VanderPlas, D. Laxalde, J. Perktold, R. Cimrman, I. Henriksen, E. A. Quintero, C. R. Harris, A. M. Archibald, A. H. Ribeiro, F. Pedregosa, P. van Mulbregt; SciPy 1.0 Contributors, SciPy 1.0: Fundamental algorithms for scientific computing in Python. *Nat. Methods* **17**, 261–272 (2020).

45. P. R. Burkholder, N. H. Giles, Jr., Induced biochemical mutations in *Bacillus subtilis*. *Am. J. Bot.* **34**, 345–348 (1947).

46. T. J. Kenney, C. P. Moran, Jr., Organization and regulation of an operon that encodes a sporulation-essential sigma factor in *Bacillus subtilis*. *J. Bacteriol.* **169**, 3329–3339 (1987).
